# Utilizing Curtailed Wind and Solar Power to Scale Up Electrolytic Hydrogen Production in Europe

**DOI:** 10.1021/acs.est.4c10168

**Published:** 2025-02-11

**Authors:** Alissa Ganter, Tyler H. Ruggles, Paolo Gabrielli, Giovanni Sansavini, Ken Caldeira

**Affiliations:** † Institute of Energy and Process Engineering, ETH Zurich, Zurich 8092, Switzerland; ‡ Department of Global Ecology, Carnegie Institution for Science, Stanford, California 94305, United States

**Keywords:** electrolytic hydrogen, electricity curtailment, hard-to-abate industry, green hydrogen, decarbonization, ammonia industry, refining industry, energy system optimization

## Abstract

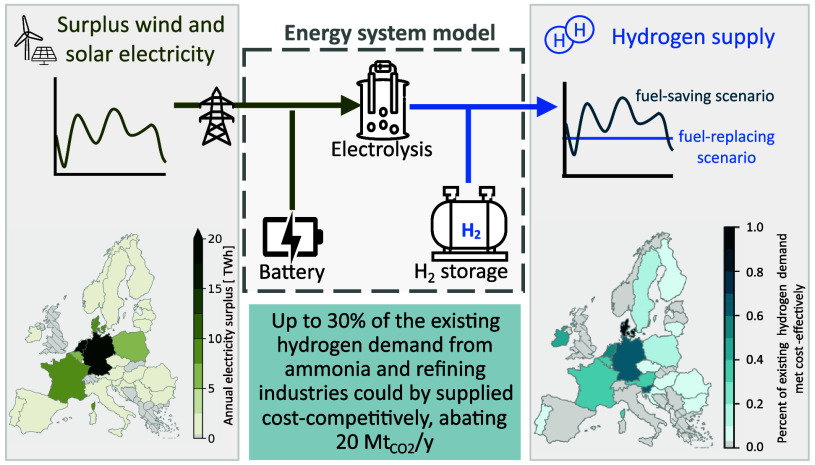

The growth of European wind and solar power capacity is associated with increasing electricity curtailment to manage excess generation and ensure safe network operations. Instead, this surplus electricity could be used to produce hydrogen, thereby reducing the need for fossil-fueled hydrogen production in ammonia and refining industries. Based on historical data, we estimate the potential for surplus electricity from wind and solar power for 27 countries across Europe. Following an optimization-based approach, we determine the cost-optimal design and operation of a system producing hydrogen from surplus electricity, including the option of battery and hydrogen storage. Two potential applications are analyzed: (1) a fuel-saving scenario, where electrolytic hydrogen substitutes fossil-fuel-derived hydrogen, whenever surplus electricity is available, and (2) a fuel-replacing scenario, where hydrogen from surplus electricity fully replaces a subset of fossil-fueled hydrogen production facilities. Our findings suggest that hydrogen from surplus electricity could substitute 30% (1.9 Mt_H2_/y) of fossil hydrogen, reducing ammonia and refinery emissions by 18% (20 Mt_CO2_/y). However, fully replacing fossil-fueled hydrogen production facilities increases hydrogen production costs substantially as it requires costly battery and/or hydrogen storage capacity to balance hydrogen production and supply. Nonetheless, about 19% (1.2 Mt_H2_/y) of the fossil-fueled hydrogen production could be replaced cost-effectively.

## Abstract 2

Electrolytic hydrogen from otherwise curtailed wind and solar power could substitute up to 30% of existing fossil-fueled hydrogen production for ammonia and refining industry in Europe cost-competitively.

## 1 Introduction

Renewable fuels, such as hydrogen from water-electrolysis by renewable electricity, are key in reducing the dependence on fossil fuels and mitigating climate change.^1^ Low-carbon hydrogen is viewed as a promising decarbonization option, particularly in sectors that inherently rely on hydrocarbon feedstocks and for which direct electrification is technologically limited or costly.^2,3^ Transitioning away from fossil fuels, however, requires substantial low-carbon hydrogen production capacity.

To date, hydrogen production is largely based on fossil fuels, such as natural gas and coal, and contributes about 3% (1.2 Gt_CO2eq_/y) of global greenhouse gas emissions.^4,5^ Scaling up low-carbon hydrogen production capacity presents an opportunity to reduce fossil hydrogen production and its associated emissions.^6,7^ Despite the rapid growth of the global electrolysis capacity in recent years, their capacity remains small (<1 GW), and accounts for less than 0.1% of the existing global hydrogen production capacity.^4^ While the number of announced electrolyzer projects is growing (∼600 projects in 2023 that are planned to be online by 2030), only about 4% of the projects reach the stage of final investment decision.^4,8^ As a result, the ambitious 2030 targets of 100 GW_el_ electrolysis capacity in the EU (∼120% of European hydrogen demand in 2022^6^), and 254 GW_el_ worldwide (∼27% of global hydrogen demand in 2022^4^), are not expected to be reached, and further highlight the persistent challenge of ramping up electrolysis capacity.^3^

Stakeholder interviews reveal that regulatory and financial challenges often cause delays.^9^ In particular, the large price differences between conventional, fossil hydrogen, and hydrogen produced from renewable electricity aggravate this problem.^10^ To derisk investments in electrolysis capacity and improve regulatory conditions are therefore crucial to enable the scale-up of the nascent low-carbon hydrogen economy.^11^

Subsidies, in the form of tax credits^12^ or fixed price premiums,^11^ are tools that can bridge the gap between conventional and electrolytic hydrogen production, thereby reducing investor risk.^13,14^ However, it has been argued that these subsidies should not extend to hydrogen produced from carbon-intensive grid electricity, which could lead to increased emissions possibly larger than those of fossil-fueled hydrogen production routes.^15^ In October 2023, the European Union (EU) therefore published two delegated acts, outlining the conditions under which renewable fuels of nonbiogenic origin (RFNBO), which include electrolytic hydrogen, are considered “renewable”.^16^ For grid-connected electrolyzers, the guidelines require a monthly balancing of electricity use with renewable electricity purchases to ensure that the additional electricity demand from hydrogen production does not increase fossil electricity generation. Ideally, the additional electricity demand introduced by electrolyzers should trigger the expansion of renewable electricity generation capacity. Temporal balancing of renewable electricity may be omitted only if electrolyzers are powered with stored, renewable electricity or otherwise curtailed electricity. For electricity prices lower or equal to 20 €/MWh the EU considers the temporal balancing fulfilled.^16^

Integrating increasing shares of variable renewable electricity generation is challenging,^17^ and countries with large shares of wind and solar capacity report increasing amounts of curtailment to ensure safe network operations.^18−20^ Nonetheless, to maximize carbon reduction, curtailment of the available renewable assets should be limited.^21^ Expanding electricity storage and transmission grid capacity, and including flexible loads, such as electrolyzers, have proven to be effective measures to reduce curtailment and foster the integration of larger shares of renewables.^22,23^

Otherwise curtailed, surplus electricity is often available at little to no cost,^24−26^ reducing the operational costs of electrolyzers. Operating electrolyzers with surplus electricity is therefore viewed as a promising solution to increase the cost-competitiveness of electrolytic hydrogen.^23,25,27^ Park et al. identify cost-optimal system configurations to produce hydrogen from surplus wind and solar power in South Korea, resulting in levelized cost of about 5.7 €/kg_H2_. Similarly, Liponi et al.^24^ and Grube et al.^28^ analyze the potential to produce hydrogen from surplus electricity in Italy and Germany with levelized costs ranging from 2.5 to 6.7 €/kg_H2_ and from 3.6 to 5.8 €/kg_H2_, respectively.

Due to the intermittent and variable supply of surplus electricity, electrolyzers often operate considerably below 100% capacity.^24,25^ This results in low capacity factors of ∼25%.^28^ Liponi et al.^24^ find that substituting surplus electricity with grid-electricity increases capacity factors and reduces hydrogen production costs. To ensure that the produced hydrogen still qualifies as an RFNBO, temporal balancing would be required whenever electricity prices exceed 20 €/MWh.^16^

Historically, fossil-fueled hydrogen production has been substantially cheaper than electrolytic hydrogen production, with costs ranging between 1.2 and 1.8 €/kg_H2_ compared to between 3.9 and 16.4 €/kg_H2_, respectively.^6,29^ Currently, hydrogen in Europe is therefore predominantly produced via steam methane reforming (SMR) of natural gas (91%), and only small shares are supplied from chemical plants that produce hydrogen as a byproduct, e.g., from ethylene or styrene production (8%), and other production processes (<1%).^30^ A surge in energy prices following the Russian invasion of Ukraine caused hydrogen production costs in the EU to skyrocket (5.7 €/kg_H2_ in 2022 vs 1.4 €/kg_H2_ in 2020^6^). Although hydrogen production costs have stabilized since (3.5 €/kg_H2_ in 2023), the EU is committed to reduce the dependency on fossil fuels and phase out natural gas.^6^ To achieve this, the EU set an ambitious target to renewably produce 10 Mt_H2_ of hydrogen per year by 2030.^1^

Harnessing otherwise curtailed, surplus electricity thus presents a unique opportunity to produce electrolytic hydrogen cost-competitively. By replacing fossil hydrogen with hydrogen from surplus electricity, ammonia plants and refineries can substantially reduce their direct process emissions without the need to build out the infrastructure that would be required when entirely depending on hydrogen from renewable electricity. Especially in the near term, this approach could present a promising opportunity to scale up electrolytic hydrogen production and enable the transition from fossil to low-carbon hydrogen. However, there has not heretofore been a comprehensive assessment of the potential to produce electrolytic hydrogen from surplus electricity available in the near term in Europe.

This work investigates the cost-competitiveness of electrolytic hydrogen production from otherwise curtailed surplus electricity to decarbonize existing hydrogen demands, which are largely attributed to ammonia production and refineries (>80%).^6^ While existing analyses use future scenarios of the German^27,31^ and Italian electricity sector^24^ and deploy energy system optimization models to estimate surplus electricity, the estimates presented in this work are based on renewable electricity generation data from recent years. A linear optimization model is deployed to assess the cost-optimal system configurations and operations to produce hydrogen from the estimated surplus electricity. In addition to electrolyzers, battery and hydrogen storage options are considered to help balance electricity and hydrogen supply.

Two hydrogen supply scenarios are considered assuming (i) very flexible or (ii) completely inflexible fossil-fueled hydrogen production. Although existing SMR facilities are often designed to operate with limited flexibility, recent evidence suggests that they can reduce their load to ∼60% and 70% of their nominal production capacity.^32,33^ Therefore, it can be expected that existing SMR facilities could operate between these two extreme scenarios.

The paper is structured as follows. Section 2 provides an overview of the considered energy system, the optimization model, and the approach used to estimate surplus electricity generation in Europe based on historical data. Sections 3 and 4 present and discuss the results.

## 2 Methodology

This work uses an optimization-based approach to determine the optimal system configuration for each country to supply electrolytic hydrogen from surplus electricity. Section 2.1 introduces the energy system model and the modeling scenarios considered in this work. Moreover, Section 2.2 provides an overview of the optimization problem. Finally, Section 2.3. describes the approach used to estimate renewable electricity curtailment in Europe.

### 2.1 System Description and Scenario Definition

**Figure 1:**
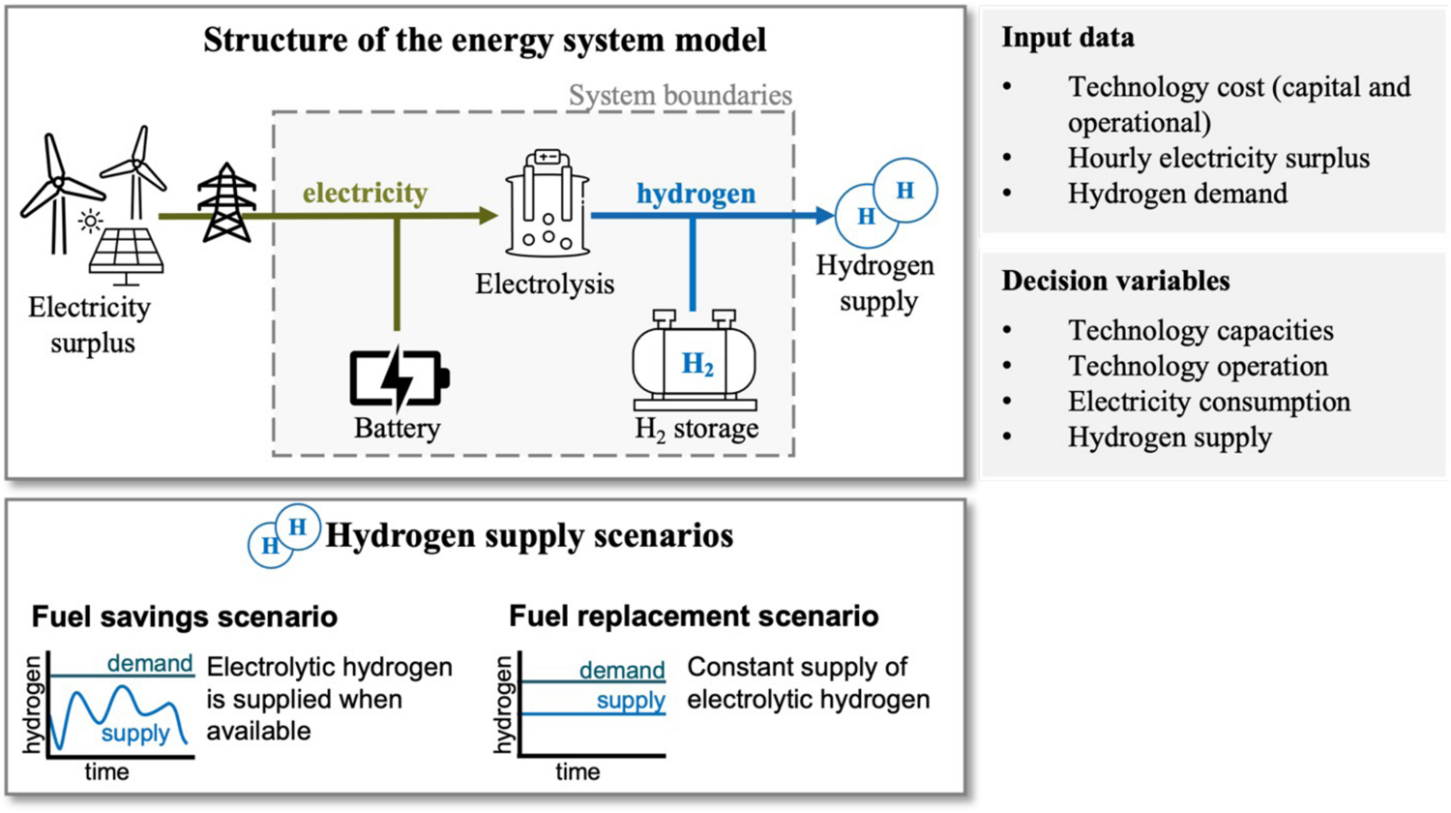
Overview of the structure of the energy system model, the input data, and the decision variables. The optimization problem minimizes total system cost and determines the optimal electrolyzer and storage capacity and dispatch depending on the available electricity surplus and the hydrogen supply scenario. The hydrogen supply scenarios are visualized below. Two hydrogen supply scenarios are considered: The fuel-savings scenario, where electrolytic hydrogen can be supplied whenever available to reduce existing fossil-fueled hydrogen production. In the fuel-replacement scenario, electrolytic hydrogen must be supplied at an hourly constant rate to replace a fixed share of the fossil-fueled hydrogen production. In both scenarios, the hourly hydrogen supply is limited by the existing hydrogen demand.

Figure 1 provides an overview of the energy system model, the input data, and the decision variables. Decision variables include electrolyzer, battery, and hydrogen storage capacity, as well as their hourly dispatch. Considering the near-term perspective of this analysis, a current state-of-the-art alkaline electrolyzer is modeled following Fonseca et al.^6^. Lithium-ion (Li-ion) batteries with storage durations of 1, 2, and 4 h can be installed to store surplus electricity for powering electrolyzers at a later time.^34^ Furthermore, the option to store hydrogen in underground pipes is included.^35^ Underground pipe storage has successfully been used for seasonal natural gas storage in Germany and Switzerland and may also be suitable for hydrogen storage. The technology presents a cost-effective alternative to gas storage tanks when salt caverns are unavailable. An additional advantage of using underground pipes is the protection from weather impacts.^35^ The cost of hydrogen compression is included in the capital and operational expenditures of the underground pipe storage technology. The techno-economic parameters of the electrolyzer, battery, and hydrogen storage technologies are summarized in Tables S1, S2, and S3, respectively.

Surplus electricity is assumed to be available at zero cost as energy-intensive industries, such as ammonia plants and refineries, have access to the intraday market in Europe.^36^ On the intraday market, electricity is traded up to 5 min before delivery.^37^ The electricity price on the intraday market is determined following the “pay-as-bid” principle.^38^ To reduce surplus and prevent overloads in times of excess generation, electricity is often offered at zero or negative prices.^39^

For each country, the system design and cost are investigated for two hydrogen supply scenarios, (1) the fuel-saving and (2) the fuel-replacing scenario (Figure 1, bottom). The fuel-saving scenario considers the production and supply of electrolytic hydrogen whenever surplus electricity is available. In the fuel-saving scenario, hydrogen supply is thus only limited by the availability of surplus electricity and the country-specific hydrogen demands from ammonia plants and refineries (Table S4).^40^ This scenario assumes completely flexible operation of the existing SMR plants. Therefore, no constraints are placed on the ramp rates of SMR facilities, i.e., for how quickly and to what degree they can change their hydrogen outputs in response to the modeled electrolytic hydrogen production from surplus electricity.

Conversely, the fuel-replacing scenario considers fully replacing a share of the existing fossil-fueled hydrogen production capacity used to supply hydrogen for ammonia production and refineries with hydrogen from surplus electricity. This scenario assumes completely inflexible operations of the existing SMR plants, therefore requiring a constant supply of hydrogen. Thus, hydrogen must be supplied at a constant hourly rate, up to the existing, country-specific hydrogen demands.

These two scenarios provide limiting cases for the use of electrolytic hydrogen, assuming it either (i) reduces very flexible fossil-fueled hydrogen production (fuel-saving scenario), or (ii) replaces a share of completely inflexible fossil-fueled hydrogen production (fuel-replacing scenario). Although large industrial plants, such as ammonia facilities or refineries, are typically operated at high loads and shut down only for maintenance, it has been noted that SMR facilities can reasonably turn down their operations to ∼60% and 70% of their nominal production capacity.^32,33^ Thus, existing fossil-fueled hydrogen production facilities would likely be able to operate between these two extreme cases.

### 2.2 Optimization Problem

The optimization problem is implemented in the optimization framework ZEN-garden (Zero-emissions Energy Networks), developed at the Reliability and Risk Engineering Lab at ETH Zurich,^7,41^ and solved using the commercial solver Gurobi^42^. The following briefly introduces input data, decision variables, constraints, and the optimization problem’s objective function. A full description of the optimization problem and its input data is provided in Supporting Information S1 and S2.

#### 2.2.1 Input Data

The input data includes the hourly availability of surplus electricity per country, the country-specific existing hydrogen demands from ammonia production and refineries, the price for supplying hydrogen, and the techno-economic parameters describing the cost and performance of electrolyzer, Li-ion batteries, and the hydrogen storage technology. Surplus electricity is available at zero cost. The hourly availability of surplus electricity is estimated based on historical capacity and generation data of wind and solar generation capacity from 2020 to 2021 for each of the 27 countries.^43^Section 2.3. describes in detail how the surplus electricity estimates are obtained.

#### 2.2.2 Decision Variables

The optimization problem determines the cost-optimal electrolyzer, battery, and hydrogen storage capacity and their hourly operations, i.e., the optimal electricity inputs and hydrogen outputs of the electrolyzers, and the optimal storage charge and discharge of battery and hydrogen storages at every hour for the different hydrogen supply scenarios. The hydrogen supply scenarios considered are introduced in Section 2.1.

#### 2.2.3 Constraints

Mass and energy balances for hydrogen and electricity must be satisfied. The electrolyzer can only be powered by surplus electricity. In addition, hydrogen supply is constrained by the existing hydrogen demands of each country. Depending on the hydrogen supply scenario, additional constraints may apply that limit the hydrogen supply (Section 2.1). Moreover, technology constraints are added to model the behavior of electrolyzer, battery, and hydrogen storage technologies.

#### 2.2.4 Objective Function

The objective function minimizes the annual system cost of one year, comprising the annualized capital expenditures and the operational expenditures of the electrolyzers, Li-ion battery, and hydrogen storage technologies and the revenue stream from supplying hydrogen over one year. The cost-optimal system configurations are investigated for a range of hydrogen prices, and incentivize production and supply of electrolytic hydrogen to reduce existing fossil-fueled hydrogen production.

### 2.3 Estimating Surplus Electricity in Europe

Today, substantial amounts of renewable electricity are curtailed when renewable electricity generation exceeds electricity demands or to solve grid congestion issues. Instead of being curtailed, this surplus electricity could be used to produce hydrogen via water-electrolysis. This work quantifies the amount of renewable electricity that is currently curtailed, i.e., the amount of surplus electricity, across the 27 EU member states. The amount of surplus electricity is estimated using the historical capacity and generation data for onshore wind, offshore wind, and solar PV that is published on the ENTSO-E Transparency Platform.^43^ The capacity and generation data are available at the national level, and with annual and hourly resolutions, respectively. The quality of the historical data from the ENTSOE-E Transparency Platform is discussed in Supporting Information S3.1.

The surplus electricity, *S*_*n,g,t*_, is estimated based on the reported actual generation, *P*_*n,g,t*_, and the estimated potential electricity generation, *P*_*n,g,t*_^max^. For each European member state, *n* ∈ *N*, technology type *g* ∈ {Solar PV, Onshore Wind, Offshore Wind}, and hour of the year, *t* ∈ *T* = {1,...,8760}, it follows:
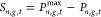
1

The potential electricity generation, *P*_*n,g,t*_^max^, of each member state *n* and hour t is estimated based on the installed technology capacity, *C*_*n,g*_, and the corresponding capacity factor, *f*_*n,g,t*_:
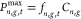
2

The capacity factor, *f*_*n,g,t*_, describes the amount of power that can be generated by technology *g* during hour *t* relative to the available technology capacity. The historical capacity factors are taken from the Copernicus climate change service operational energy data set.^44^ The capacity factors are estimated based on climate data from the ERA5 global reanalysis climate data set, which includes temperature, surface solar radiation, and wind speed data.

## 3 Results

### 3.1 Surplus Electricity in Europe

**Figure 2:**
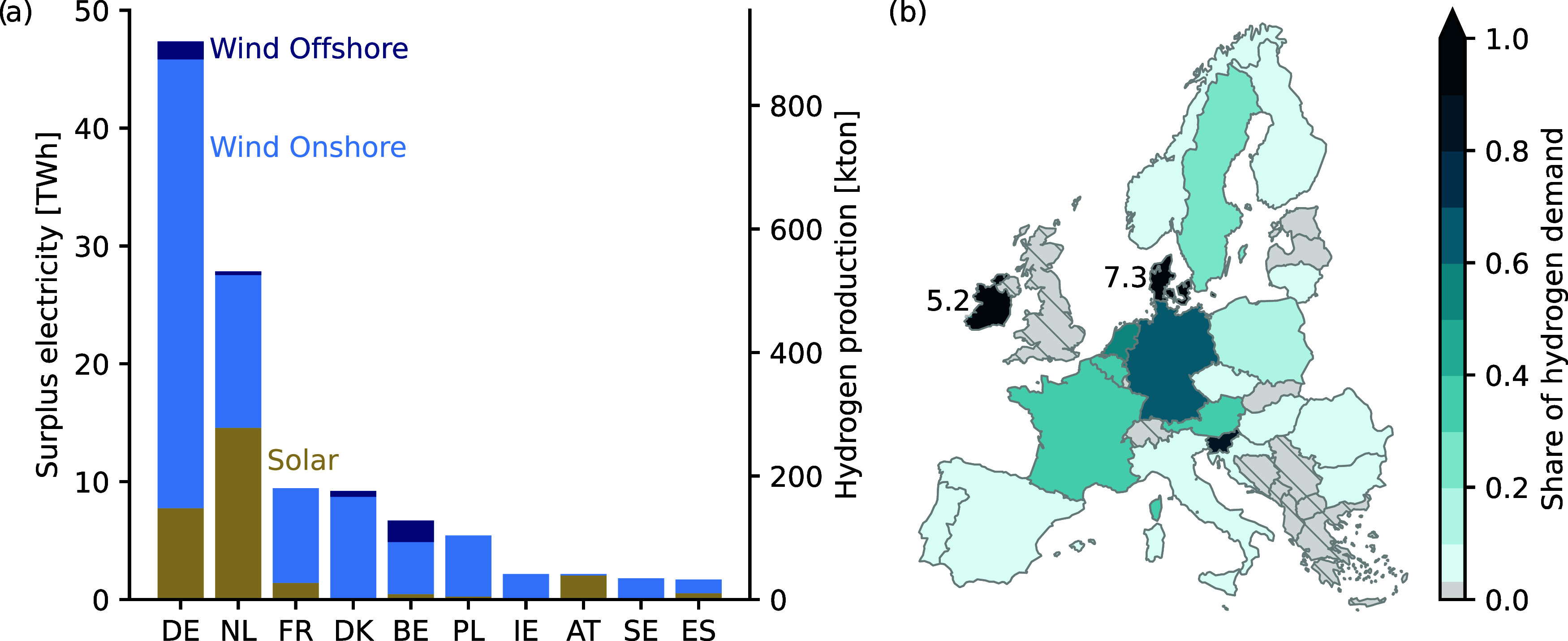
(a) Surplus electricity per technology type and potential hydrogen production for the ten European countries with the largest availability of surplus electricity in 2022. (b) Share of hydrogen demand that can be met utilizing 100% of the available surplus electricity. For countries shaded in solid gray, no hydrogen demand from ammonia production and refineries is reported (i.e., Latvia, Estonia, and Slovenia). The hydrogen demand values are reported in Table S4.

Figure 2a shows the surplus electricity from wind onshore, wind offshore, and solar photovoltaics (PV) for the top ten European countries in 2022. The results for the years prior to 2022 are reported in Supporting Information S3.2.

Between 2020 and 2022, about 95 to 134 TWh of renewable electricity was curtailed in Europe each year, which corresponds to between 3% to 4% of the annual European electricity demand.^45^ This likely represents a conservative estimate of renewable electricity curtailment as our analysis is limited by country-level electricity generation and capacity data, which underestimates the renewable electricity generation potential of the individual assets (Supporting Information S.3.1).

Curtailment affects onshore wind power plants the most, followed by solar PV (72% of the estimated renewable electricity curtailment in 2022 was from wind power plants and 24% from solar PV). The amount of renewable electricity curtailment per technology is strongly related to the renewable electricity generation potential. Although the existing generation capacity for onshore wind and solar PV is similar (50% and 45% of total existing renewable electricity generation capacity, respectively), the renewable electricity generation potential for solar PV is much lower compared to that of onshore wind power plants (142 TWh for solar versus 383 TWh for onshore wind in 2022). Moreover, the available electricity generation capacity is heterogeneously distributed across countries and depends on the regional renewable electricity generation potentials from wind and solar. Therefore, large differences are observed in the generation types that are curtailed at the country level. For instance, the Netherlands curtails similar shares of solar PV (53%) and wind (47%), whereas Belgium predominantly curtails wind power and Austria predominantly curtails solar PV. Moreover, an analysis of the periodicity of the hourly time series suggests strong daily patterns for solar-PV-dominated countries, while for wind-dominated countries, seasonal patterns are dominant (Figure S6).

Figure 2b shows the share of hydrogen demand that countries can meet by utilizing 100% of their surplus electricity to produce hydrogen. Assuming an electrolyzer efficiency of 52.4 kWh_el_/kg_H2_, up to 36% of the annual European hydrogen demands for ammonia production and refineries could be supplied. Germany (DE) and the Netherlands (NL) show the largest hydrogen production potential from surplus electricity and could meet 68% and 60% of their existing hydrogen demands, respectively. Denmark (DK) is also among the top five countries with the largest hydrogen production potential in Europe. However, compared to Denmark’s hydrogen production potential, existing hydrogen demands are small (24 kt_H2_/y versus 176 kt_H2_/y). This would allow Denmark to consistently meet its existing hydrogen demands with hydrogen from surplus electricity while exporting about 152 kt_H2_/y to neighboring countries such as Germany, where several refineries are located in close proximity to the Danish border, e.g., in Hamburg, Wilhelmshaven, or Bremen.^46^ Ireland’s hydrogen demands are also small compared to its hydrogen production potential (8 kt_H2_/y versus 41 kt_H2_/y), enabling Ireland to act as a hydrogen exporter as well. Other potential exporting countries are Estonia, Latvia, and Luxembourg, for which currently no existing hydrogen demands are reported. In comparison to Denmark, however, their hydrogen production potential is small (from 2 to 9 kt_H2_/y). Rather than export this hydrogen, it could be used to advance the decarbonization of other industries that rely on hydrogen in their decarbonization strategies, such as steel and cement industries.^2^

### 3.2 Utilizing Hydrogen from Surplus Electricity: Fuel-Saving vs Fuel-Replacing

The availability of surplus electricity varies largely across countries. At first, the focus of the analysis is on Germany, the Netherlands, Belgium, and Austria, which are among the countries with the largest hydrogen production potentials. Combined, the four countries could supply up to 1.5 Mt_H2_/y of hydrogen when utilizing 100% of the surplus electricity (56% of their current hydrogen demand^40^).

**Figure 3:**
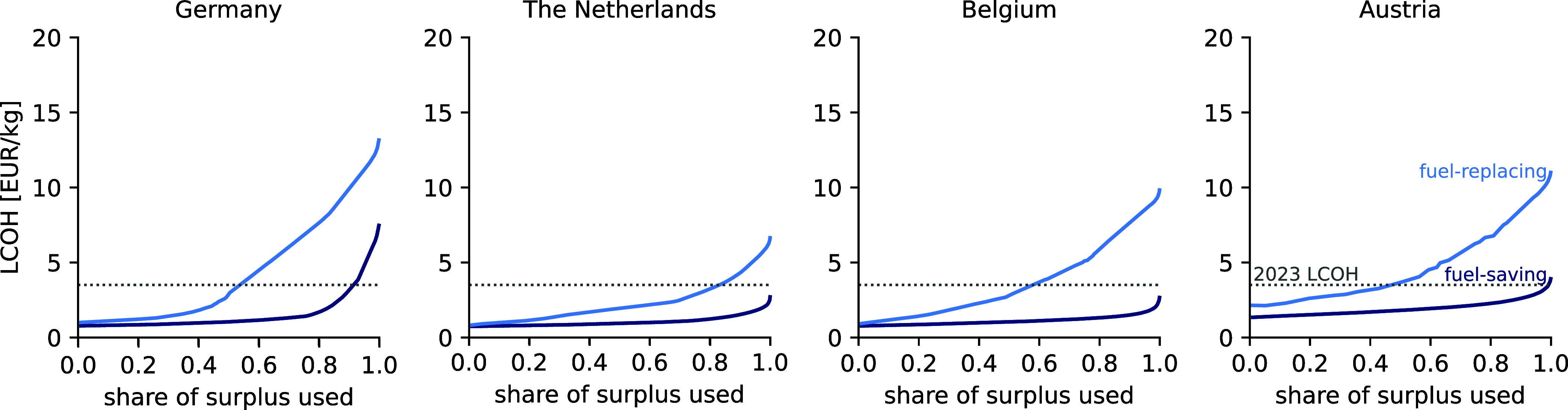
Levelized cost of hydrogen (LCOH) as a function of the share of surplus electricity used in the fuel-saving scenario (dark blue) and the fuel-replacing scenario (light blue). The gray dotted line indicates the average LCOH for fossil-fueled hydrogen production from 2023.^6^ The fuel-saving scenario considers the substitution of fossil-fueled hydrogen production with electrolytic hydrogen production whenever surplus wind and/or solar electricity is available. In the fuel-replacing scenario, hydrogen produced from surplus electricity must be supplied at hourly constant rates, thus requiring the installation of a battery and/or hydrogen storage that results in substantially higher LCOH compared to the fuel-saving scenario.

Accessing increasing shares of surplus electricity increases the levelized cost of hydrogen (LCOH) as it requires the installation of a larger electrolyzer and/or the addition of energy storage. Figure 3 shows the LCOH as a function of the share of surplus electricity that is used for Germany, the Netherlands, Belgium, and Austria. For reference, the gray dotted line indicates the average LCOH for fossil-fueled hydrogen production in the EU, which was at 3.5 €/kg_H2_ in 2023. For a comprehensive overview of the results for the remaining countries, refer to Supporting Information S4.

Both hydrogen supply scenarios are shown: (1) the fuel-saving scenario (dark blue) and (2) the fuel-replacing scenario (light blue). In the fuel-saving scenario, a moderate increase in the LCOH is observed when utilizing increasing shares of surplus electricity (<90%). However, accessing shares above 90% often substantially increases cost as it implies the utilization of surplus electricity during peak hours, which requires (i) substantially larger electrolysis capacity and/or (ii) the installation of battery storage capacity to store excess electricity and use it later.

**Figure 4:**
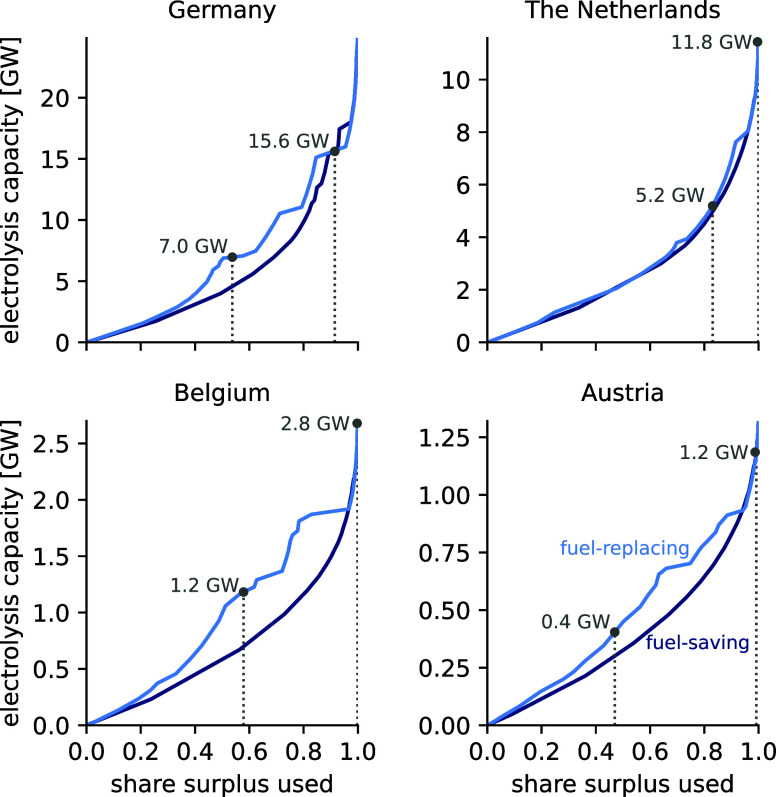
Electrolysis capacity as a function of the share of surplus electricity used in the fuel-saving scenario (dark blue) and the fuel-replacing scenario (light blue). The fuel-saving scenario considers the substitution of fossil-fueled hydrogen production with electrolytic hydrogen production whenever surplus wind and/or solar electricity is available. In the fuel-replacing scenario, hydrogen produced from surplus electricity must be supplied at hourly constant rates, thus requiring the installation of a battery and/or hydrogen storage. In most countries, hydrogen storage is installed, and the electrolysis capacity is increased to store hydrogen when excess surplus electricity is available and discharge hydrogen during hours when less or no surplus electricity is available. The gray dotted line indicates the capacity and share of surplus electricity used when the levelized cost corresponds to the average levelized cost for fossil-fueled hydrogen production reported in 2023 (3.5 €/kg_H2_).^6^

Figure 4 shows the optimal electrolysis capacity as increasing shares of surplus electricity are utilized. In general, expanding electrolysis capacity proves more cost-effective than the installation and operation of expensive battery storage capacity (Figure S8). Only for countries where surplus electricity is particularly volatile such as Italy or Hungary (i.e., high coefficient of variation), is it cost-effective to install and operate battery storage capacity to balance the electricity supply and increase electrolysis capacity factors (Figure S11). In contrast, the addition of hydrogen storage is only needed if hydrogen production exceeds hydrogen demand when large shares of surplus electricity are utilized (>90%, Figure S10).

In the fuel-replacing scenario, the LCOH of the selected countries is between two and three times higher than in the fuel-saving scenario. Due to the requirement to provide a constant hydrogen supply, larger electrolyzers and hydrogen storage capacity are installed than for comparable amounts of hydrogen in the fuel-saving scenario. Hydrogen storage is filled during hours of sufficient availability of surplus electricity and discharged during hours where little or no surplus electricity is available to balance hydrogen production and achieve a constant hydrogen supply. Again, the addition of battery storage remains limited to cases where the variability in the surplus electricity is particularly high (e.g., Italy (IT) and Hungary (HU), Figure S4c), and/or when accessing large shares of surplus electricity (>80%).

A comparison of the LCOH across countries shows that the LCOH is generally lower in countries with higher availability of surplus electricity. Nonetheless, this is not a guarantee to produce hydrogen cost-effectively. Besides the total amount of surplus electricity, the variability of the surplus electricity should be considered (Figure S5). If the variability is high, large battery storage capacity is needed to balance the intermittent electricity supply and maintain sufficient electrolysis capacity factors (e.g., Italy). Alternatively, large electrolysis capacity would need to be installed, but would also remain unused for most of the time (e.g., Spain). Both options, the addition of energy storage or the installation of larger electrolysis capacity, significantly increase capital expenditures and lead to high LCOH (compare Italy and Spain (ES) in Figures S7 and S9). Even when considering a 25% reduction in battery storage cost, hydrogen storage remains the preferred storage option (Supporting Information 4.7). Hence, for electrolytic hydrogen to become cost-effective in countries with high variability in surplus electricity, substantial reductions of electrolyzer and battery storage costs are required.

### 3.3 Cost-Competitive Hydrogen Production from Surplus Electricity in Europe

**Figure 5:**
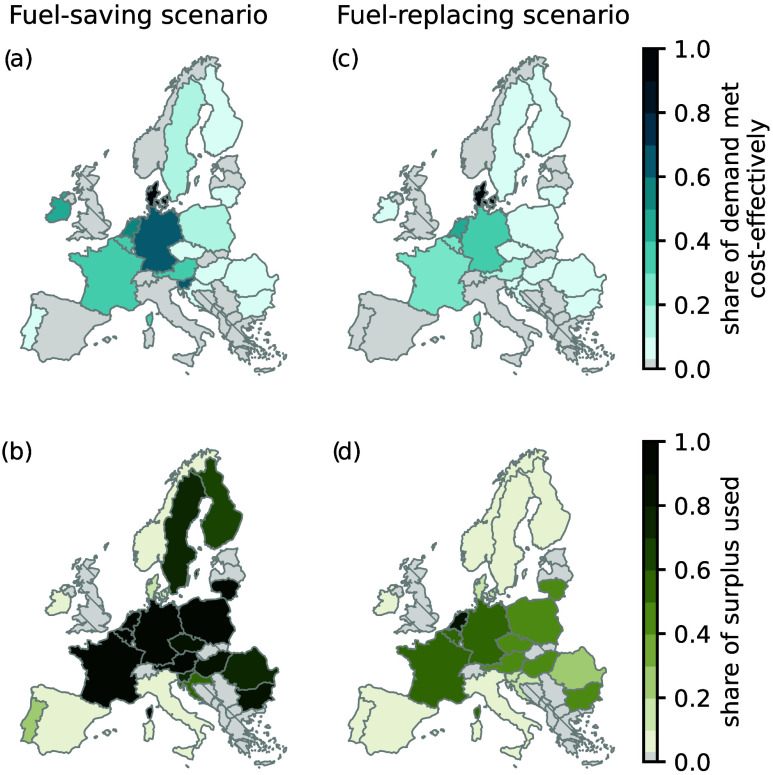
Share of hydrogen demand that can be met cost-competitively by utilizing surplus electricity when considering the average levelized cost for fossil-fueled hydrogen production from 2023 (top row), and the share of surplus electricity used (bottom row) in the fuel-saving scenario (left column) and the fuel-replacing scenario (right column). The hydrogen demand for each country is reported in Table S4. Compared to the fuel-saving scenario, the share of hydrogen demand that can be met cost-effectively reduces in the fuel-replacing scenario as costs increase due to the installation of larger electrolyzers and the addition of battery and/or hydrogen storage.

The cost-competitiveness of producing hydrogen from surplus electricity in the fuel-saving and the fuel-replacing scenarios is evaluated, considering the average LCOH for fossil-fueled hydrogen production from 2023 of 3.5 €/kg_H2_.^6^Figure 5a shows the share of hydrogen demand that the fuel-saving scenario can meet cost-competitively, and Figure 5b shows the corresponding share of surplus electricity that is utilized in the fuel-saving scenario.

The results indicate that the fuel-saving scenario could satisfy about 30% of the existing fossil hydrogen demands cost-effectively, utilizing 85% of the available surplus electricity. The average carbon intensity of hydrogen production from SMR is 10.5 kg_CO2_ per kg hydrogen produced.^6^ This 30% reduction in fossil-fueled hydrogen production would, thus, equate to an 18% (20 Mt_CO2_) reduction of ammonia and refinery emissions and incentivize the installation of 42 GW_el_ electrolyzers across Europe. The utilization of energy storage options remains limited and is only used when hydrogen supply would otherwise exceed demands (e.g., in Germany or France).

Figure 5c shows the share of hydrogen demand that could be fulfilled by utilizing the available surplus electricity (Figure 5d) to supply hydrogen in the fuel-replacing scenario, considering the average LCOH of fossil-based hydrogen production from 2023 (3.5 €/kg _H2_).^6^ Due to the higher LCOH in the fuel-replacing scenario, the amount of hydrogen demand that can be replaced cost-effectively reduces to 19% (-11% compared to the fuel-saving scenario). Overall, this would require an electrolysis capacity of approximately 17 GW_el_, which corresponds to about 40% of the electrolysis capacity that would be installed in the fuel-saving scenario. Moreover, about 47 kt_H2_ hydrogen storage is needed to balance hydrogen production and supply. While the addition of battery storage capacity would enable a steadier electricity supply, and increase electrolyzer full load hours, a very large battery storage capacity would be required. As a result, battery storage options remain cost-inefficient in most cases. Even when considering a 25% reduction in battery storage costs, the installation of hydrogen storage in combination with a larger electrolysis capacity is preferred (Supporting Information S4.7). Although the cost of storing hydrogen is generally higher than for electricity in terms of €/MWh, smaller storage volumes are required upon conversion.

Compared to the fuel-saving scenario, substantially lower shares of the available surplus electricity are utilized cost-effectively in the fuel-replacing scenario (Figure 5d), and about 46% of the surplus electricity remains unused. Lower-cost battery and hydrogen storage technologies will help diminish the gap in the LCOH and the level of surplus electricity cost-effectively utilized between the fuel-saving and the fuel-replacing scenarios.

Here, the average LCOH from 2023 for fossil-fueled hydrogen production is used as a reference (3.5 €/kg_H2_ assuming a natural gas price of 50 €/MWh).^6^ However, large variations in hydrogen production costs have been observed in recent years in the EU. For instance, the natural gas shortage following the Russian invasion of Ukraine in 2022 led to elevated LCOH, and the EU average increased to 5.7 €/kg_H2_. When considering the elevated LCOH of 2022 as a reference (Table S4), the amount of hydrogen that can be produced cost-competitively from surplus electricity increases by up to 15%. While the LCOH has since stabilized around 3.5 €/kg_H2_, historically, values from 1.8 to 2.6 €/kg_H2_ were reported.^6^ Even when considering LCOH as low as 2.2 €/kg_H2_, between 14% and 28% of the existing hydrogen demands can be fulfilled cost-competitively in the fuel-replacing and the fuel-saving scenarios, respectively.

In addition to the variability in the natural gas price, volatile carbon prices impact the LCOH of fossil hydrogen. Assuming an average carbon price of 85 €/ton_CO2_, increases the LCOH by 0.55 €/kg_H2_.^6^ Within the European Emission Trading System, the share of carbon emission allowances is reduced on an annual basis in line with EU climate targets.^47^ As a result, the carbon price is expected to increase in the future. Nonetheless, compared to the natural gas price, the impact of increasing carbon prices on the LCOH of fossil hydrogen is small. Assuming a natural gas price of 50 €/MWh, the carbon price would need to increase to 425 €/t_CO2_ to observe a similar impact on the LCOH as that of the high natural gas prices in 2022.

### 3.4 Exporting Hydrogen from Surplus Electricity to Neighboring Countries

Hydrogen production from surplus electricity exceeds the existing hydrogen demands of Denmark and Ireland, enabling both countries to export hydrogen to regions of neighboring countries. Besides Ireland and Denmark, Estonia, Luxembourg, and Latvia may act as hydrogen exporters as they currently do not report any existing hydrogen demands. However, compared to Denmark, their hydrogen production potentials are small (from 2 to 9 kt_H2_/y versus 140 kt_H2_/y).

To investigate the potential of Denmark, Ireland, Estonia, and Luxembourg to export hydrogen, we remove the constraint that ensures that hydrogen supply does not exceed the existing hydrogen demand of the individual countries. As before, two hydrogen supply scenarios are considered, one where hydrogen supply is unconstrained (fuel-saving), and one where hydrogen must be supplied at hourly constant rates (fuel-replacing).

**Figure 6:**
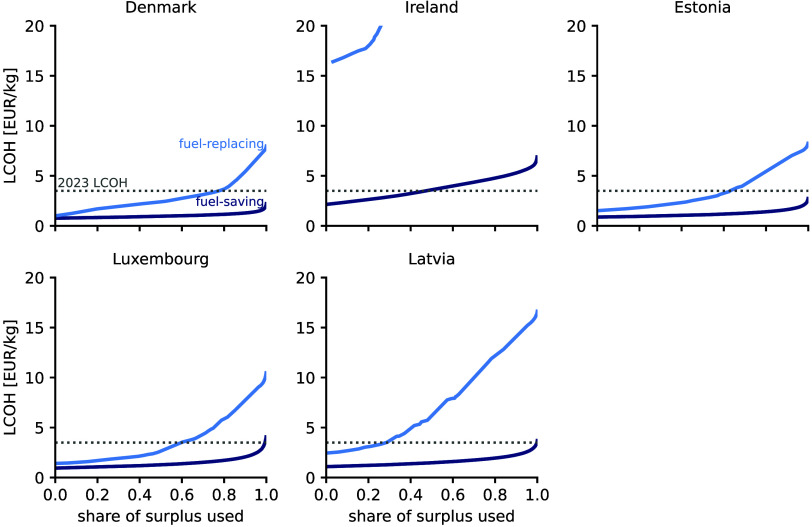
Levelized cost of hydrogen (LCOH) as a function of the share of surplus electricity used when hourly hydrogen supply is unconstrained (fuel-saving scenario, dark blue), and when hydrogen has to be supplied at a constant hourly rate (fuel-replacing scenario, light blue) in countries with the potential to export hydrogen from surplus wind and solar electricity as local hydrogen production would otherwise exceed their existing demands. The gray dotted line indicates the country-level LCOH reported in 2023.^6^ The requirement to supply hydrogen at a constant rate in the fuel-replacing scenario requires the installation of hydrogen and/or battery storage and thus increases cost substantially as larger shares of surplus electricity are used.

Figure 6 shows the LCOH depending on the share of surplus electricity used for the two delivery scenarios: (1) the fuel-saving scenario (dark blue) and (2) the fuel-replacing scenario (light blue). Furthermore, the gray dotted line indicates the average LCOH for fossil-fueled hydrogen production in 2023. In the fuel-saving scenario, up to 90% of the renewable surplus can be utilized cost-competitively (considering the average LCOH from 2023), and about 195 kt_H2_/y hydrogen can be supplied to neighboring countries. However, when considering a lower LCOH of 2.2 €/kg_H2_, the export potential reduces by 19% to 158 kt_H2_/y.

In the fuel-replacing scenario, export potentials reduce to 152 kt_H2_/y of hydrogen, 80% of which is produced in Denmark. Despite the potential to produce 41 kt_H2_/y of hydrogen in Ireland, the high variability of the surplus electricity (Figure S4c) leads to high LCOH, and hydrogen production in Ireland remains unattractive from a cost perspective in this scenario. For reduced LCOH of 2.2 €/kg_H2_, export potentials further reduce to 44 kt_H2_/y, and only Denmark remains cost competitive.

## 4 Discussion

### 4.1 Scaling Up Electrolytic Hydrogen Production Utilizing Surplus Electricity

European electrolysis capacity currently stands at 2.9 GW_el_ (2023).^6^ To meet the EU’s ambitious targets to produce 10 Mt_H2_/y renewable hydrogen by 2030, an electrolysis capacity of 100–120 GW_el_ is required.^6^ However, an IEA analysis^4^ reveals that projects often fail in the final stage of investment decisions due to financial barriers.

Almost 80% of the announced electrolyzer projects in Europe plan to use electricity from dedicated renewables.^8^ While increasing carbon prices can help reduce the price gap between renewable and fossil hydrogen, in the short term, carbon prices from 700 to 1300 €/t_CO2_ would be needed to achieve cost-competitiveness.^48^ Moreover, the hydrogen production cost varies significantly depending on the location. For instance, hydrogen production costs are up to 25% lower in solar-rich countries such as Portugal and Spain (from 5.2 to 5.8 €/kg_H2_) than in Germany or the Netherlands (from 7.3 to 7.68 €/kg_H2_).^6^

Besides financial barriers, limited electrolyzer manufacturing capacity, uncertainty on the future low-carbon hydrogen demand, and the development of a hydrogen infrastructure present barriers and hinder the ramp-up of electrolysis capacity. For instance, Europe’s existing electrolyzer manufacturing capacity would need to grow by at least 40% per year to meet the electrolysis capacity target and supply 10 Mt_H2_/y of renewable hydrogen by 2030.^6^

Considering conventional technology expansion rates, the EU will likely not achieve its 2030 renewable hydrogen production target, and breakthroughs before 2040 are not expected^3^ without the introduction of policy instruments to help bridge this gap and create business cases to support investments in renewable hydrogen production capacity.^11^ In March 2024, the EU therefore introduced the concept of the European Hydrogen Bank, which aims to provide financial support to scale up domestic renewable hydrogen production.^49^

Utilizing existing surplus electricity could provide another business case to ramp up electrolysis capacity and mitigate scarce low-carbon hydrogen supply in the near term. About 1.65 Mt_H2_/y of fossil hydrogen could be substituted by hydrogen from surplus electricity and facilitate investments in 42 GW_el_ electrolysis capacity. These investments would substantially ramp up existing electrolysis capacity and facilitate cost reductions due to learning effects and economies of scale in the medium and long-term.^48^

High amounts of surplus electricity combined with large existing hydrogen demands make Germany, the Netherlands, and Belgium particularly attractive locations for electrolyzer investments (Figure 5). Using surplus electricity to produce hydrogen in these same countries may be promising also because the hydrogen production cost from dedicated renewable electricity generation capacity remains high.^50^ Nonetheless, only about 10% of the announced electrolyzer projects currently consider a connection to the electricity grid and could, thus, have access to low-cost surplus electricity.

To fully replace existing hydrogen produced from fossil resources with hydrogen produced from surplus electricity presents substantial challenges for two reasons. First, ammonia plants will need to undergo retrofitting to fully replace SMR with electrolysis. Besides modifications of the existing equipment, this entails the installation of air separation units to produce nitrogen for ammonia synthesis, which is currently also supplied by the integrated SMR process. Without retrofitting, the use of electrolytic hydrogen is limited to ∼15%^51^, across scenarios. Although the anticipated retrofitting costs are small compared to the hydrogen production cost (5–10% of ammonia production cost compared to 90–95%),^32^ retrofitting requirements must be considered when planning the transition to net-zero emission ammonia production. Nonetheless, utilizing surplus electricity presents a unique opportunity to reduce the use of fossil hydrogen for ammonia production in the near term.

Second, the industry processes that rely on hydrogen as a feedstock typically require a constant supply of hydrogen and have been designed with limited flexibility. To achieve an uninterrupted hydrogen supply from surplus electricity, therefore, the installation of costly energy storage solutions, such as battery or hydrogen storage systems, is necessary to manage the intermittent nature of surplus electricity from wind and solar PV. Similar to Stolte et al.^52^, it is observed that hydrogen storage options are generally preferred over battery storage options. However, these additional storage requirements lead to a substantial increase in the overall hydrogen production cost and reduce the cost-competitiveness. Despite these challenges, there are cost-effective opportunities to expand electrolysis capacity by up to 17 GW_el_, contribute to the transition toward renewable hydrogen production, and support the development of sustainable hydrogen infrastructure.

### 4.2 Renewable Electricity Curtailment due to Grid-Congestion Issues

With the continuous expansion of renewable electricity generation capacity, increasing wind curtailment rates due to grid congestion issues are reported across Europe.^53^ To limit renewable curtailment due to grid-congestion issues and foster the successful integration of larger shares of renewables, an expansion of the existing transmission and distribution grid capacity is required. While expanding the existing distribution and transmission grid infrastructure is an effective measure to reduce curtailment, grid modernization and expansion projects often take years to plan and are capital-expensive.^54^ The IEA,^54^ therefore, strongly recommends that governments support grid expansion projects to successfully integrate the growing shares of renewables and achieve their decarbonization targets. Besides grid expansion, flexible loads, such as electrolyzers, are shown to reduce renewable curtailment and enable high utilization rates for existing renewable assets.^23^ However, this requires electrolyzers to be colocated with renewable assets, often resulting in low electrolysis capacity factors.^26^

Here, central electrolysis capacities are considered to utilize the available surplus electricity at a country level. The amount of usable surplus electricity, therefore, might reduce by 0.3 to 6.7% when considering electricity curtailment due to grid congestion issues.^53^ To fully access the untapped potential of low-cost, surplus electricity, and maximize the use of existing renewable assets, investments in grid-infrastructure projects would be required such that curtailment due to grid congestion issues is minimized.

### 4.3 Cost-Competitiveness of Electrolytic Hydrogen and Hydrogen Production Coupled with Carbon Capture and Storage

Currently, hydrogen is produced predominantly via SMR of natural gas.^6^ On average, this process emits 10.5 kg_CO2_/kg_H2_. To reduce process emissions, SMR can be coupled with carbon capture and storage (CCS). According to the RFNBO regulations, the process qualifies as “low carbon” if emissions remain below 3.4 kg_CO2_/kg_H2_.^55^ However, to achieve emission levels comparable to electrolytic hydrogen, very high carbon capture rates (>90%) and low methane leakage rates (<1%) are required.^56,57^

Despite the substantial cost-reduction potential for electrolyzers, studies often find SMR coupled with CCS to be more cost-competitive.^29,48,58^ Especially in regions where natural gas prices are below 15 €/MWh, electrolytic hydrogen is not envisioned to become cost-competitive before 2040.^48^

The hydrogen production cost of SMR-CCS strongly depends on the natural gas price and the carbon transport and storage costs, both of which are subject to uncertainty. In particular natural gas prices can be very volatile.^59^ For instance, the exceptionally high natural gas prices in the year 2022 increased hydrogen production costs by over 200% and caused almost half of the European ammonia producers to suspend production.^60^ Currently, Northern Lights in Norway is the only operational carbon storage site in Europe, with one more under construction.^23,61^ While the capacity of the announced carbon storage projects put the EU on track to meet its medium-term goal, the plans to ramp up carbon injection capacity are ambitious, considering the long planning and construction times for carbon storage projects.^62^ This limited availability of carbon transport and storage capacity in the near term will likely reflect in cost with estimates ranging from 4 €/t_CO2_ up to 45 €/t_CO2_.^63^ As a result, the range of the hydrogen production costs for SMR-CCS is large (from 4.4 to 10 €/kg_H2_).^6^

In addition to SMR-CCS, methane pyrolysis could be considered as a means to produce low-carbon hydrogen. The process yields hydrogen and solid carbon via an endothermic reaction that splits methane into its components. Hydrogen production costs range from 2.3 to 5.9 €/kg_H2_, depending on the natural gas price and the additional revenue stream that can be generated from selling solid carbon.^64^

In comparison, the hydrogen production cost from dedicated renewable assets is estimated at ∼7 €/kg_H2_ (European average).^6^ Utilizing surplus electricity, which is often available at little to no cost, could thus provide a promising avenue to scale up low-carbon hydrogen production in the near term. These investments in electrolyzers will help drive economies of scale and increase the cost-competitiveness of electrolytic hydrogen production in the medium and long term. Coupling existing SMR capacity with CCS can provide a transitional solution to reduce emissions and mitigate stranded assets in the near term. Moreover, methane pyrolysis may be a promising alternative in countries where natural gas prices are low and where the availability of surplus electricity is limited. This alternative could prolong the use of natural gas, counteract the EU targets to reduce the dependency on natural gas imports, and further delay the scale-up of electrolyzers.^3^

### 4.4 Central Model Assumptions and Limitations

A state-of-the-art alkaline electrolyzer is considered for hydrogen production. Although proton exchange membrane electrolyzers offer greater flexibility, alkaline electrolyzers are currently more cost-competitive. Moreover, by operating the modular stacks of the alkaline electrolyzers separately from one another, the flexibility of the system can be increased.^33^

Due to the national resolution, grid constraints that may limit the amount of surplus electricity that is accessible by individual hydrogen production facilities are not represented in detail. Nonetheless, low curtailment rates due to grid congestion issues suggest that over 90% of the surplus electricity may be accessible.^53^ Assuming that each ammonia and refinery plant has access to the national surplus electricity proportionally to its hydrogen demand translates into assuming that individual facilities install electrolyzer, battery, and hydrogen storage capacity corresponding to their hydrogen demand. Alternatively, our modeling assumptions can be seen as centralized electrolyzer, battery, and hydrogen storage installation at the national level, where the hydrogen transport to the individual hydrogen production facilities is not modeled. However, transporting electricity is generally more cost-effective.^65^

Conversely, a more detailed representation of the electricity grid constraints would require spatially resolved data on the existing renewable generation capacity as well as the actual generation per generation unit of each country. Although per-generation-unit data is published by the ENTSO-E for some countries,^43^ it is not available for all considered countries, and time series are often incomplete. Therefore, the country-level capacity and generation data is used in this analysis. The quality of the data retrieved from the ENTSO-E Transparency Platform is discussed in more detail in Supporting Information S3.1.

It is assumed that surplus electricity is available at zero cost. While this is a common assumption in literature, when electricity would otherwise remain unused (e.g., refs (24−26)), increasing competition for surplus electricity might increase costs in the long term. In addition, grid fees from the transmission system operators may apply as only a few countries currently waive the grid fees for electrolyzers (e.g., via the Energy Industry Act in Germany^66^ or the Federal Electricity Supply Act in Switzerland^67,68^). Considering electricity prices of up to 20 €/MWh_el_ reduces the cost-competitiveness of electrolyzers. Nonetheless, about 15% to 28% of the annual hydrogen demands could be met cost-effectively in the fuel-replacing and the fuel-saving scenarios, respectively. To hedge against uncertain future electricity prices, power purchase agreements are needed.

Here, two hydrogen supply scenarios are presented: (1) the fuel-saving scenario, and (2) the fuel-replacing scenario. Possible ramping constraints for existing SMR facilities are not modeled. Currently, most plants are designed to operate at a steady load.^32^ The requirement to supply hydrogen at constant rates (fuel-replacing scenario), therefore, may be closer to what is technically feasible. However, recent analyses suggest that SMR facilities could operate with some limit flexibility.^33^ Future work could therefore investigate in more detail the technical constraints of flexible operation of SMR facilities and analyze the potential of using hydrogen as a fuel-saving to support the transition to hydrogen produced with low carbon emissions in ammonia production and refiners.

This analysis focuses on decarbonizing existing hydrogen demands in the near-term. Although hydrogen demand is projected to grow, its application in industry is expected to evolve. As the demand for liquid fuels decreases, the hydrogen demand from refineries is anticipated to decline. In contrast, the hydrogen demands for ammonia production are expected to remain stable. In addition, new hydrogen demands might emerge to enable the decarbonization of methanol, steel, and cement production. Although hydrogen is envisioned to play a central role in decarbonizing industrial processes, global production and trade patterns may change due to the heterogeneous availability of renewable resources.^48^ Investments in hydrogen transport infrastructure can help mitigate the risk of industry relocation and enable the transport of hydrogen between current and future demand locations.

While this work focuses on utilizing surplus electricity for hydrogen production, future research should also explore competing applications for surplus electricity in terms of cost and their potential emission abatement. Besides producing electrolytic hydrogen, battery storage could be installed to counteract short-term imbalances and support the decarbonization of the power sector.^69^ The cost of battery storage has drastically reduced in recent years,^70^ and recent findings indicate that performing electricity arbitrage with Li-ion battery storage is economically attractive in most European countries.^71^

Building on this work, future research could also investigate in more detail electricity grid constraints or potential hydrogen transport requirements considering the individual plant locations for ammonia production and refineries. We are not suggesting that businesses and governments base infrastructure investment decisions on our study alone; rather, the results indicates that businesses and governments could benefit from more careful consideration of particular infrastructure investments that might cost-effectively produce green hydrogen by taking advantage of wind and solar power that would otherwise be curtailed.

### 4.5 Concluding Remarks

With growing shares of renewable wind and solar PV generation capacity in Europe, the amount of electricity curtailment is expected to increase. Instead of being curtailed, this surplus electricity could be used to produce hydrogen. Already today, the utilization of surplus electricity could result in a significant amount of electrolytic hydrogen being produced cost-competitively with hydrogen produced from fossil fuels. The results presented here indicate that the ammonia and refining industries in Europe could cost-effectively substitute up to 30% of their currently fossil-fueled hydrogen production with electrolytic hydrogen derived from surplus electricity (fuel-saving scenario), reducing their existing emissions by 18% (20 Mt_CO2_). Supplying hydrogen at hourly constant rates (fuel-replacing scenario) would require the addition of costly hydrogen storage capacity. In this case, there is less opportunity for ammonia and refining industries to produce electrolytic hydrogen cost-competitively due to the increased system costs. Nonetheless, ammonia and refining industries could replace up to 19% of their existing fossil-fueled hydrogen production cost-effectively, while reducing their existing emissions by 11% (13 Mt_CO2_).

The results further suggest that Germany and the Netherlands are among the countries with the largest potential for hydrogen production from surplus electricity. Considering average hydrogen production costs from 2023, both countries can meet up to 60% of their existing hydrogen demands cost-competitively, utilizing over 90% of their available surplus electricity. Even when considering a constant hydrogen supply, ammonia and refining industries in Germany and the Netherlands could meet 36% and 50%, respectively, of their existing hydrogen demands cost-competitively. The high local natural gas prices and large existing hydrogen demands make electrolyzer projects utilizing surplus electricity in Germany and the Netherlands particularly interesting; and offer large emission reduction potentials in the near term.

Moreover, countries where the potential for green hydrogen production from surplus electricity exceeds their existing demands, such as Denmark or Latvia, could act as green hydrogen exporters in the near future. Alternatively, excess green hydrogen could be used to advance decarbonization in other sectors and industries (e.g., steel and cement industries). The green hydrogen export potential is estimated at 152 kt_H2_, over 90% of which would be produced in Denmark.

In conclusion, using hydrogen produced from surplus wind and solar power to substitute for hydrogen produced from fossil fuels is a promising approach for scaling up electrolysis capacity in the near term. The results indicate to EU policymakers that this could cost-effectively provide up to 19% of the EU’s renewable hydrogen production target for 2030. To do so would require a 42 GW_el_ increase in electrolysis capacity. Moving from substituting for fossil hydrogen to fully replacing a share of the existing fossil-fueled hydrogen production facilities would require electricity or hydrogen storage to ensure uninterrupted hydrogen supply and balance variable surplus electricity. While there is some limited potential for fuel replacement using hydrogen from surplus electricity today, scaling up such replacement would likely require cost reductions in energy storage technologies and/or additional dispatchable carbon-emission-free generation capacity.

## Data Availability

The data and code underlying this study are openly available in the following Zenodo repository: 10.5281/zenodo.13827117.

## Supporting Information Available

Input data to the optimization problem; mathematical formulation of the optimization problem; additional information on the estimation of surplus electricity based on historical electricity generation data; additional figures visualizing the levelized cost of hydrogen, the electrolysis capacity, the hydrogen and battery storage capacity across the considered countries depending on the share of surplus that is used; storage cost sensitivity (PDF)
