# Supplementary material for: Utilizing Curtailed Wind and Solar Power to Scale Up Electrolytic Hydrogen Production in Europe
Source: Environ Sci Technol. 2025 Feb 11;59(7):3495–507. doi: 10.1021/acs.est.4c10168 (PMC11866934; doi:10.1021/acs.est.4c10168)
Supplement: Supplementary file 1 — es4c10168_si_001.pdf [file es4c10168_si_001.pdf]

Supporting Information for

Utilizing curtailed wind and solar power to scale up electrolytic hydrogen production in Europe

Alissa Ganter[1,2], Tyler H. Ruggles[2], Paolo Gabrielli[1,†], Giovanni Sansavini[1,*], Ken Caldeira[2]

[1] Institute of Energy and Process Engineering, ETH Zurich, Zurich 8092, Switzerland

[2] Department of Global Ecology, Carnegie Institution for Science, Stanford, CA 94305, USA

** Corresponding author: Giovanni Sansavini (sansavig@ethz.ch)*

[†] Current affiliation: Huawei Technologies Switzerland, Zurich Research Center, Zurich 8050, Switzerland

Summary: 19 pages, 7 tables, 15 figures.

S1. Input data to the optimization problem

Section S1.1. reports the technology parameters for the electrolyzer, the battery, and hydrogen storage options. Section S1.2. reports the existing hydrogen demands for each country and specifies the levelized cost of hydrogen reported in 2022.

S1.1. Technology parameters

Table S1, S2, and S3 provide an overview of the techno-economic input data used to model the electrolyzer, battery and hydrogen storage technologies, respectively.

Table S1. Techno-economic electrolyzer input data assumptions based on [1].

Parameter	Electrolyzer	Unit
Capex	1250	€/kW
Fixed opex: Stack replacement costs	438	€/kW
Fixed opex: Other	25	€/kW/y
Stack lifetime	80,000	h
Lifetime	30	y
Conversion factor	52.4	kWh/kg

Table S2. Techno-economic input data assumptions for utility-scale, standalone battery storage with storage durations 1h, 2h, and 4h based on [2].

Parameter	Battery storage 1h	Battery storage 2h	Battery storage 4h	Unit
Capex (power)	113	113	113	€/kW
Capex (energy)	336	283	279	€/kWh
Fixed opex	6	5	4	€/kW
Lifetime	20	20	20	y
Duration	1	2	4	h
Charge efficiency	0.95	0.95	0.95	-
Discharge efficiency	0.95	0.95	0.95	-
Self-discharge	0.001	0.001	0.001	-

Table S3. Techno-economic input data assumptions for hydrogen pipe storage underground based on [3].

**Parameter**	**Hydrogen pipe underground**	**Unit**
Capex (energy)	526	€/kg
Fixed opex	10	€/kg
Lifetime	30	y
Charge efficiency	0.99	-
Discharge efficiency	0.99	-
Self-discharge	0	-

S1.2. Existing hydrogen demands and levelized cost of hydrogen

Table S4. Hydrogen demands for ammonia production and refineries [4] and levelized cost of hydrogen reported for 2022 [1]. The country codes are based on the Nomenclature of Territorial Units for Statistics (NUTS) [5].

Country code	Country name	Hydrogen demand [kt]	Levelized cost [€/kg]
AT	Austria	111	6.95
BE	Belgium	347	5.15
BG	Bulgaria	118	6.68
HR	Croatia	62	4.52
CZ	Czechia	99	5.29
DK	Denmark	24	7.43
EE	Estonia	0	6.81
FI	Finland	171	7.93
FR	France	489	4.76
DE	Germany	1330	6.17
GR	Greece	326	7.12
HU	Hungary	181	6.93
IE	Ireland	8	5.33
IT	Italy	590	7.25
LV	Latvia	0	5.72
LT	Lithuania	144	7.10
NL	Netherlands	883	5.8
LU	Luxembourg	0	5.92
PL	Poland	769	4.77
PT	Portugal	97	6.02
RO	Romania	116	7.47
SK	Slovakia	103	5.13
SI	Slovenia	2	4.48
ES	Spain	563	5.39
SE	Sweden	150	9.57

S2. Optimization problem

Section S2.1. provides an overview of the parameters and decision variables of the optimization problem. The constraints of the optimization problem are reported in Section S2.2. Finally, Section S2.3. describes the objective function.

S2.1. Parameters and decision variables

The parameters and decision variables of the optimization problem are summarized in Table S5 and Table S6.

Table S5. Overview of the parameters of the optimization problem.

**Variable**	**Description**
$S_{n,t}$	available surplus electricity in country n at time t
$\mathrm{d}_{\mathrm{c,n,t}}$	demand of carrier c in country n at time t
η_g	conversion factor of conversion technology g
ζ_g	energy to power ratio of storage technology g
δ_g	self-discharge factor of storage technology g
a_g	annualization factor for storage technology g
r	discount rate
L_g	lifetime of technology g
α_g	unit cost technology g in country n
β_g^{fix}	fixed operational expenditures of technology g
p_c	price of carrier c

Table S6. Overview of the decision variables of the optimization problem.

Variable	Description
$F_{c,n,t}^{imp}$	import flow of carrier c in country n at time t
$F_{c,n,t}^{exp}$	export flow of carrier c in country n at time t
$P_{c,n,t,g}^{in}$	input flow of carrier c and conversion technology g in country n at time t
$P_{c,n,t,g}^{out}$	output flow of carrier c and conversion technology g in country n at time t
$SL_{c,n,t,g}^{+}$	storage charge of carrier c and storage technology g in country n at time t
$SL_{c,n,t,g}^{-}$	storage discharge of carrier c and storage technology g in country n at time t
$SL_{c,n,t,g}$	storage level of carrier c and storage technology g in country n at time t
$C_{n,g}$	power capacity of technology g in country n
$C_{n,g}^{\mathrm{E}}$	energy capacity of storage technology g in country n
$\mathrm{CAPEX}_{n,g}$	capital expenditures of technology g in country n
$\mathrm{OPEX}_{n,g}$	operational expenditures of technology g in country n
$\mathrm{CARR}_{\mathrm{c,n}}$	carrier cost of carrier c in country n

S2.2. Constraints

Energy balances. The energy balance for the energy carriers $c \in C$, electricity and hydrogen, must be fulfilled for each country $n \in N$ and hour $t \in T$.

$$F_{c,n,t}^{imp} - F_{c,n,t}^{exp} + P_{c,n,t,g}^{out} - P_{c,n,t,g}^{in} + SL_{c,n,t,g}^{+} - SL_{c,n,t,g}^{-} - \delta_g \, SL_{c,n,t,g} = 0 \tag{1}$$

where $F_{c,n,t}^{imp}$ and $F_{c,n,t}^{exp}$ represent the import and export flows of carrier c in country n at time t, respectively, $P_{c,n,t,g}^{in}$ and $P_{c,n,t,g}^{out}$ represent the input and output flows of carrier c and conversion technology g in country n at time t, respectively. Moreover, $SL_{c,n,t,g}^{+}$ and $SL_{c,n,t,g}^{-}$ represents the storage charge and discharge of carrier c and storage technology g in country n at time t, respectively. Lastly, $SL_{c,n,t,g}$ represents the storage level of carrier c and storage technology g in country n at time t, and δ_g represents the corresponding self-discharge factor.

Electricity (el) is the only carrier that can be imported (i.e., $F_{\mathrm{H2},n,t}^{imp}$= 0). The electricity import is limited to the available electricity surplus $S_{n,t}$:

$$F_{\mathrm{el},n,t}^{imp} \leq S_{n,t} \tag{2}$$

Hydrogen is the only carrier that can be exported (i.e., $F^{exp}_{el,n,t}$= 0), representing the hydrogen supply to industry to meet a share of the existing hydrogen demand. The hydrogen export is therefore limited by the level of the existing hydrogen demand $\mathrm{d_{H2,n,t}}$:

$$F^{exp}_{H2,n,t} \leq \mathrm{d_{H2,n,t}} \tag{3}$$

Conversion technology constraints. The technology output of a conversion technology is limited by the installed capacity $C_{n,g}$:

$$P^{out}_{c\prime,n,t,g} \leq C_{n,g} \tag{4}$$

The conversion of carriers c into carrier c' ($c \neq c$') is expressed via the conversion factor η_g:

$$P^{in}_{c,n,t,g} = \eta_g P^{out}_{c\prime,n,t,g} \tag{5}$$

Storage technology constraints. The storage charge and discharge capacity, and the storage levels are limited by the storage power and energy capacity $C_{n,g}$, and $C^E_{n,g}$, respectively:

$$SL_{c,n,t,g} \leq C_{n,g} \tag{6}$$

$$SL_{c,n,t,g} \leq C^E_{n,g} \tag{7}$$

The storage level $SL_{c,n,t,g}$ is determined based on the storage charge $SL^+_{c,n,t,g}$ and discharge $SL^-_{c,n,t,g}$, and the storage level of the previous time-step, as well as self-discharge losses:

$$SL_{c,n,t,g} = \eta^+_g SL^+_{c,n,t,g} - \eta^-_g SL^-_{c,n,t,g} - \delta_g\, SL_{c,n,t-1,g} \tag{8}$$

Furthermore, the storage power and energy capacity are linked by the energy-to-power ratio.

$$\zeta_g\, C_{n,g} = C^E_{n,g} \tag{9}$$

Fuel-saving scenario. No additional constraints are added to the optimization problem.

Fuel-replacing scenario. Hydrogen must be supplied at a constant rate. Thus, the export of hydrogen has to be constant over time:

$$F^{exp}_{H2,n,t} = F^{exp}_{H2,n,t\prime} \quad \text{where} \;\; t \neq t', \forall\, t, t' \epsilon\, T \tag{10}$$

S2.3. Objective function

The objective function minimizes the net present cost of the energy system.

$$min \sum_{n \in N} \left(\sum_{g \in \mathrm{G}} \left(a_g\, \mathrm{CAPEX}_{n,g} + \mathrm{OPEX}_{n,g} \right) - \sum_{c \in C} \mathrm{CARR_{c,n}} \right) \tag{11}$$

where $\mathrm{CAPEX}_{n,g}$ and $\mathrm{OPEX}_{n,g}$ represent the capital and operational expenditures of each technology $g\ \epsilon$ G, and $\mathrm{CARR_{c,n}}$ represents the carrier costs that incur from utilizing surplus electricity to produce and supply hydrogen. The optimization is conducted for the time horizon of 1 year with hourly resolution (i.e., 8760 time steps). Therefore, the capital expenditures are multiplied by the annualization factor a_g:

$$a_g = \frac{r\,(1+r)^{L_g - 1}}{(1+r)^{L_g} - 1} \tag{12}$$

where r represents the discount rate and L_g represents the technology lifetime. Here, we use a discount rate of 6%.

The capital expenditures of each technology are computed based on the installed capacity $C_{n,g}$ and the technology unit cost α_g. For storage technologies, an energy and power-rated unit cost is applied to the energy and power-rated storage capacity.

$$\mathrm{CAPEX}_{n,g} = \alpha_g C_{n,g} \tag{13}$$

The operational expenditures comprise fixed operational expenditures that occur e.g., due to technology maintenance. The fixed operational expenditures of each technology are computed based on the installed capacity $C_{n,g}$ and the unit cost for fixed operational expenditures β_g^{fix}. For storage technologies, energy and power-rated costs can be applied depending on the energy and power-rated storage capacity that are installed.

$$\mathrm{OPEX}_{n,g} = \beta_g^{\mathrm{fix}} C_{n,g} \tag{14}$$

Finally, the carrier costs are computed based on the costs that occur from importing and exporting carriers, i.e., from utilizing surplus electricity and from supplying hydrogen.

$$\mathrm{CARR}_{\mathrm{c,n}} = \sum_{t \in \mathcal{T}} p_c F_{c,n,t}^{imp} - p_c F_{c,n,t}^{exp} \tag{15}$$

We assume that surplus electricity is available at zero cost. Furthermore, we use a hydrogen price to incentivize the production and supply of hydrogen. Without a hydrogen price, there would be no incentive to invest in electrolysis capacity and to produce and supply hydrogen. As the hydrogen price increases, the incentive to utilize surplus electricity to produce and supply hydrogen grows. We explore how the system design changes for increasing hydrogen price as growing shares of the available surplus electricity are utilized. The cost-effectiveness of the different system designs in each country are evaluated in terms of the levelized cost of hydrogen $\mathrm{LCOH}_{\mathrm{c,n}}$:

$$\mathrm{LCOH}_{\mathrm{c,n}} = \frac{a_g \mathrm{CAPEX}_{n,g} + \mathrm{OPEX}_{n,g}}{\sum_{t \in \mathcal{T}} F_{\mathrm{H2},n,t}^{exp}} \tag{16}$$

S3. Estimating surplus electricity based on historical electricity generation data

The surplus electricity is estimated based on the renewable electricity generation capacity and the actual generation reported by the ENTSO-E Transparency Platform. The data is extracted for the three most recent years, 2020-2022. Renewable technologies include solar PV, onshore wind, and offshore wind. Section S3.1. addresses data quality issues concerning the ENTSO-E Transparency Platform. The surplus electricity estimates for 2020 and 2021 are reported in Section S3.2. Moreover, Section S3.3. reports the existing renewable electricity generation capacity. Section S3.4. analyzes the variability of the surplus electricity, and Section S3.5 compares the periodicity across different time series depending on the available technology mix.

S3.1. Data quality assessment of ENTSO-E Transparency Platform data

Data quality issues have been raised concerning the electricity generation, load, transmission, and balancing data published on the ENTSO-E Transparency Platform [6]. While data quality is high for some countries, including Austria, France, and Germany, data is inconsistent or incomplete for others, including Cyprus and Hungary. To address these quality issues, we assess the consistency and completeness of the datasets taken from the ENTSO-E Transparency Platform. If single hours are missing from the time series, the missing value is estimated by interpolating the generation from the previous and following hours. Countries missing more than 1% of the hourly generation for a technology type are excluded from further analysis. Table S7 summarizes the countries excluded from further analysis due to incomplete data. Nevertheless, the number of incomplete time series reduces throughout the years, to only 5 countries in 2022.

Table S7. Countries where the reported actual generation of solar PV, wind onshore, or wind offshore is missing more than 1% (>100 hours) of their actual generation data and which are therefore excluded from further analysis.

**Technology**	**2020**	**2021**	**2022**
Solar PV	Denmark, France, Hungary, Poland, Slovakia	Montenegro, Sweden, Ukraine	Ukraine
Wind Onshore	Denmark, Hungary, Slovakia	Bosnia Herzegovina, Montenegro, Kosovo, Ukraine	Bosnia Herzegovina, North Macedonia, Croatia, Ukraine
Wind Offshore	Denmark, Portugal	-	-

Moreover, we observe that during some hours, the reported actual generation per technology type exceeds our estimate of the potential renewable generation during that hour. In this case, we assume the available surplus electricity to be zero. This mismatch between the renewable generation estimates and actual generation occurs predominantly in hours with small capacity factors (<0.1). Our analysis relies on country-level capacity factor estimates as plant-specific electricity generation data is unavailable. These country-level capacity factors represent the average generation per technology type during each hour but do not capture the regional differences across a country. Thus, the potential electricity generation from renewables is likely underestimated, particularly when capacity factors are low. The country-level surplus electricity generation estimates, therefore, likely represent conservative estimates of the surplus electricity. Relative to the available surplus electricity of the different countries, however, these differences remain small (<0.5%).

S3.2. Surplus electricity from wind and solar across European countries in 2020 and 2021

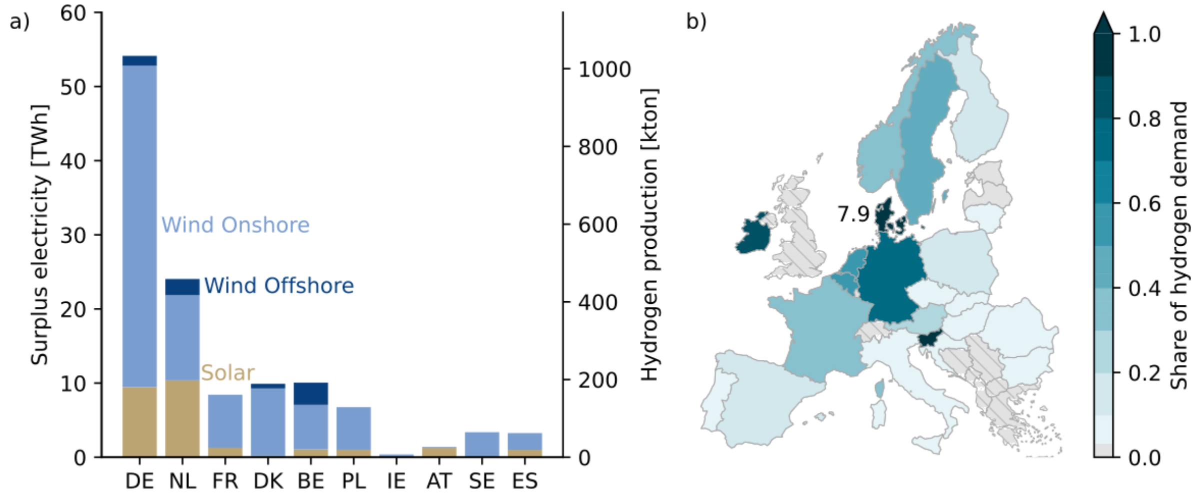

Figure S1. Surplus electricity and potential hydrogen production per technology type for the ten EU countries with the largest surplus electricity in 2020.

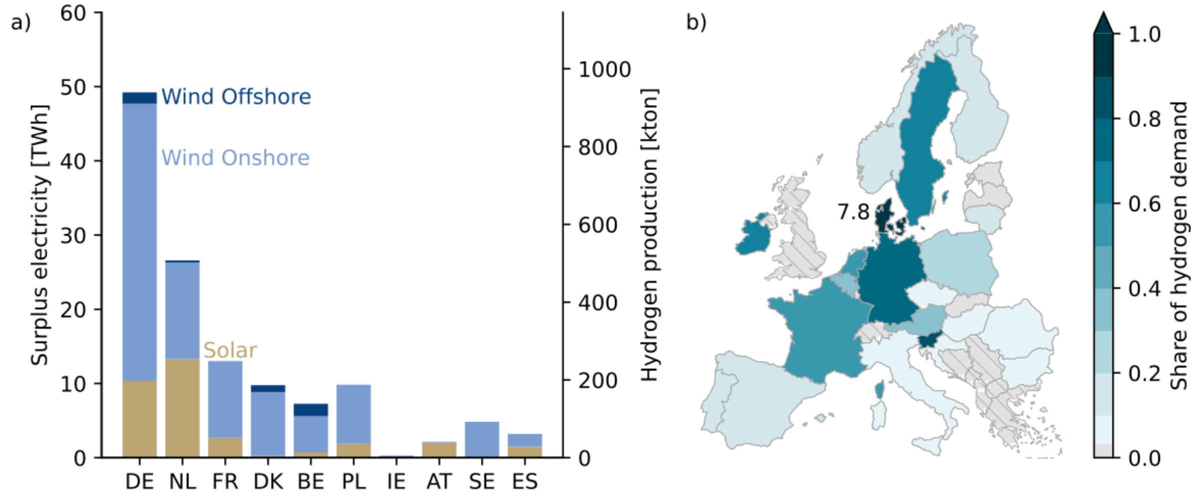

Figure S2. Surplus electricity and potential hydrogen production per technology type for the ten EU countries with the largest surplus electricity in 2021.

S3.3. Existing wind and solar electricity generation capacity across European countries from 2020-2022

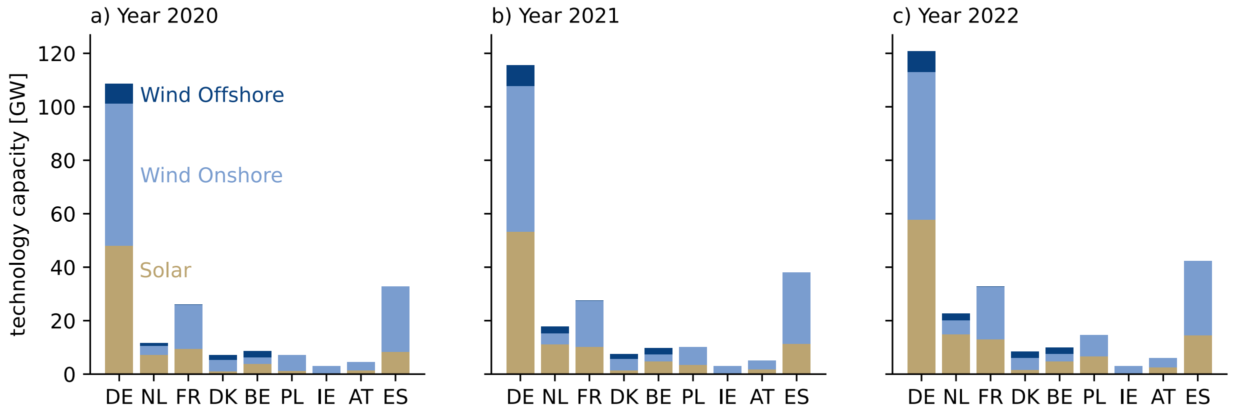

Figure S3. Existing electricity generation capacity for the ten EU countries with the largest surplus electricity for the years a) 2020, b) 2021, and c) 2022 reported on the ENTSO-E Transparency Platform [7].

S3.4. Distribution of country-level surplus electricity and coefficient of variation

Figure S3 shows the distribution of the hourly surplus electricity across the European countries in 2020-2022. In addition, we compare the mean availability of surplus renewable electricity and measure the dispersion using the coefficient of variation (i.e., the standard deviation divided by the mean) to gain more insights into the characteristics of the available surplus electricity. The comparison reveals that the dispersion is often higher in countries where less surplus electricity is available (i.e., low mean). Nonetheless, a higher availability of surplus electricity is not necessarily a guarantee for a low dispersion. Spain (ES), for example, curtails between 1.7-3.7 TWh of renewable electricity annually; but curtailment is concentrated on a few hours of the year, and on average very little surplus electricity is available (Figure S3b and Figure S4). An analysis of the periodicity of the hourly time series suggests strong daily patterns for solar-PV-dominated countries, while for wind-dominated countries, seasonal patterns are dominant (Figure S5).

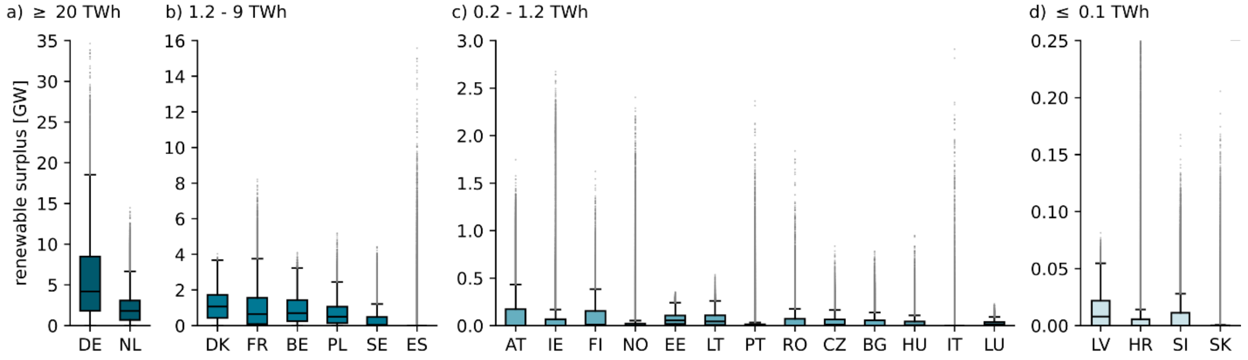

Figure S4. Distribution of hourly surplus electricity across European countries from 2020-2022. Countries are grouped based on their mean annual surplus electricity availability a) ≥ 20 TWh, b) 1.2-9 TWh, c) 0.2-1.2 TWh, and d) ≤ 0.1 TWh.

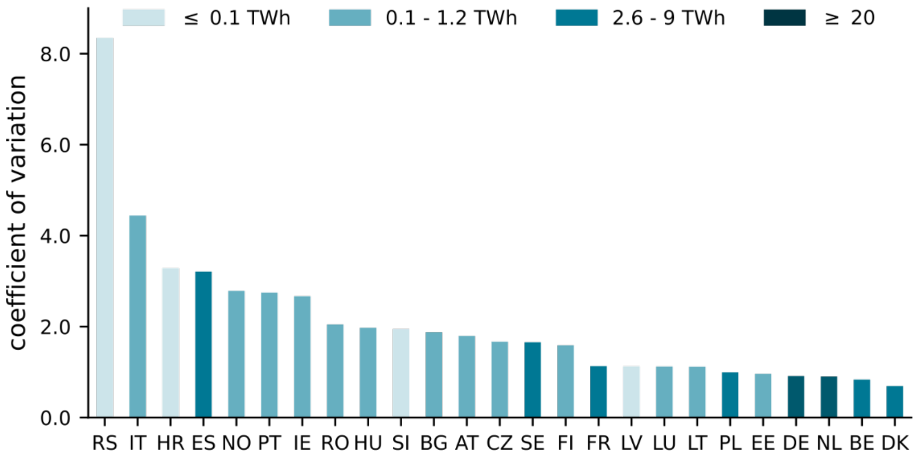

Figure S5. Coefficient of variation of the surplus electricity for each country. The coefficient of variation is a measure of the variability and is derived as the standard deviation divided by the mean. The color of the bars indicates the mean annual surplus electricity availability of each country.

S3.5. Fast Fourier Transformation of country-level surplus electricity time series

We apply a Fast Fourier Transformation to decompose the time-series in their frequency components and identify seasonality patterns. Figure S6 visualizes the country-specific time series (left) and the amplitude of the Fast Fourier Transform (right). The color reveals whether the electricity mix is wind dominated (blue) or solar dominated (bronze), i.e., over 50% of the surplus electricity is from wind or solar PV, respectively. Here we show the results for the year 2022 presented in the paper. However, the same observation is made when conducting the analysis for 2020 and 2021.

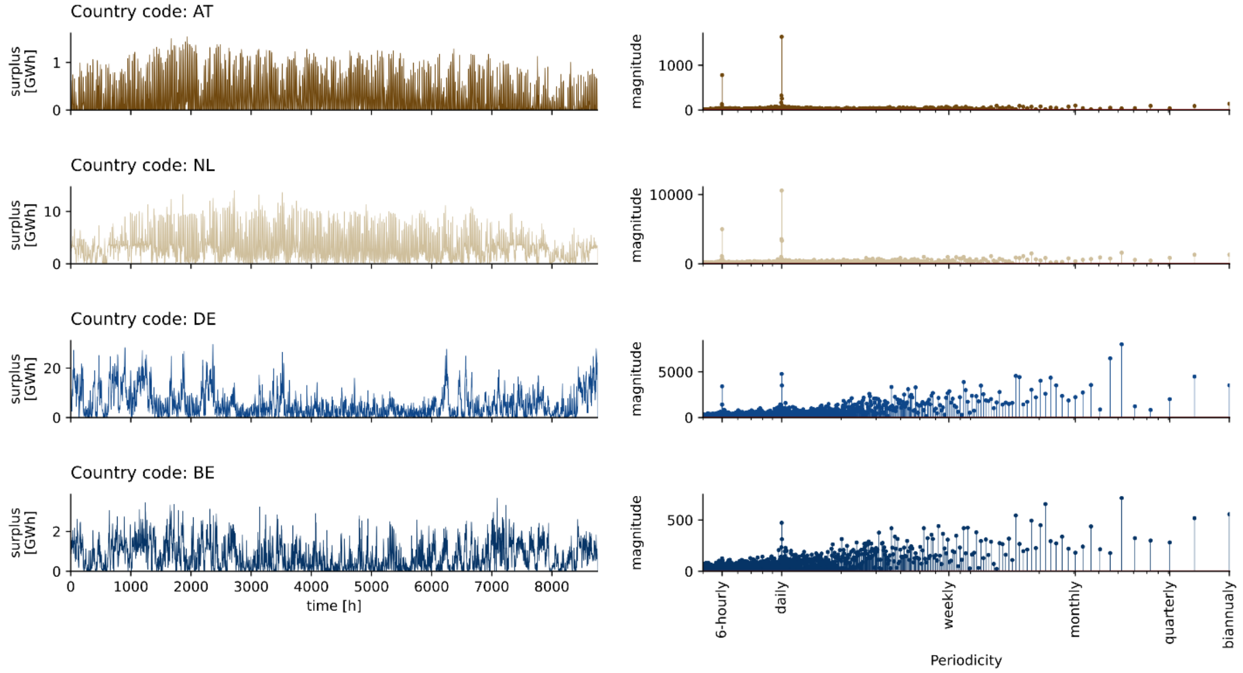

Figure S6. a) Time series of the available surplus electricity in 2022 for Austria (AT), the Netherlands (NL), Germany (DE), and Belgium (BE), and b) the results of the Fast Fourier Transform, which visualizes the amplitude and periodicity (cycles per day) for each country.

S4. Cost-optimal hydrogen production utilizing surplus electricity

S4.1. Levelized cost of hydrogen as a function of the utilization rate of surplus electricity

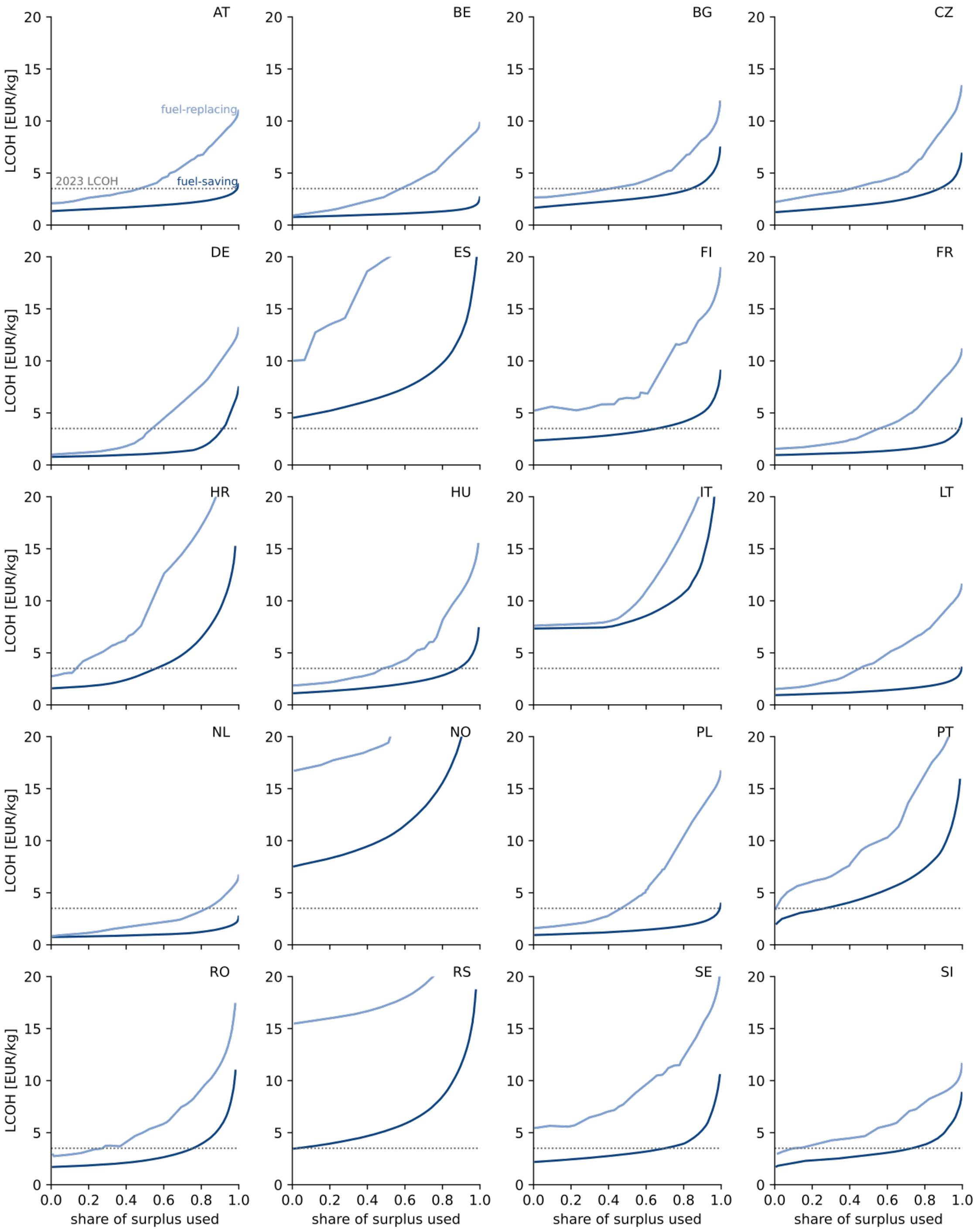

Figure S7. Country-level levelized cost of hydrogen (LCOH) in the fuel-saving scenario (dark blue) and the fuel-replacing scenario (light blue) depending on the share of surplus electricity used. The grey dotted line indicates the average LCOH for fossil hydrogen production reported in 2023 [1].

S4.2. Electrolysis capacity as a function of the utilization rate of surplus electricity

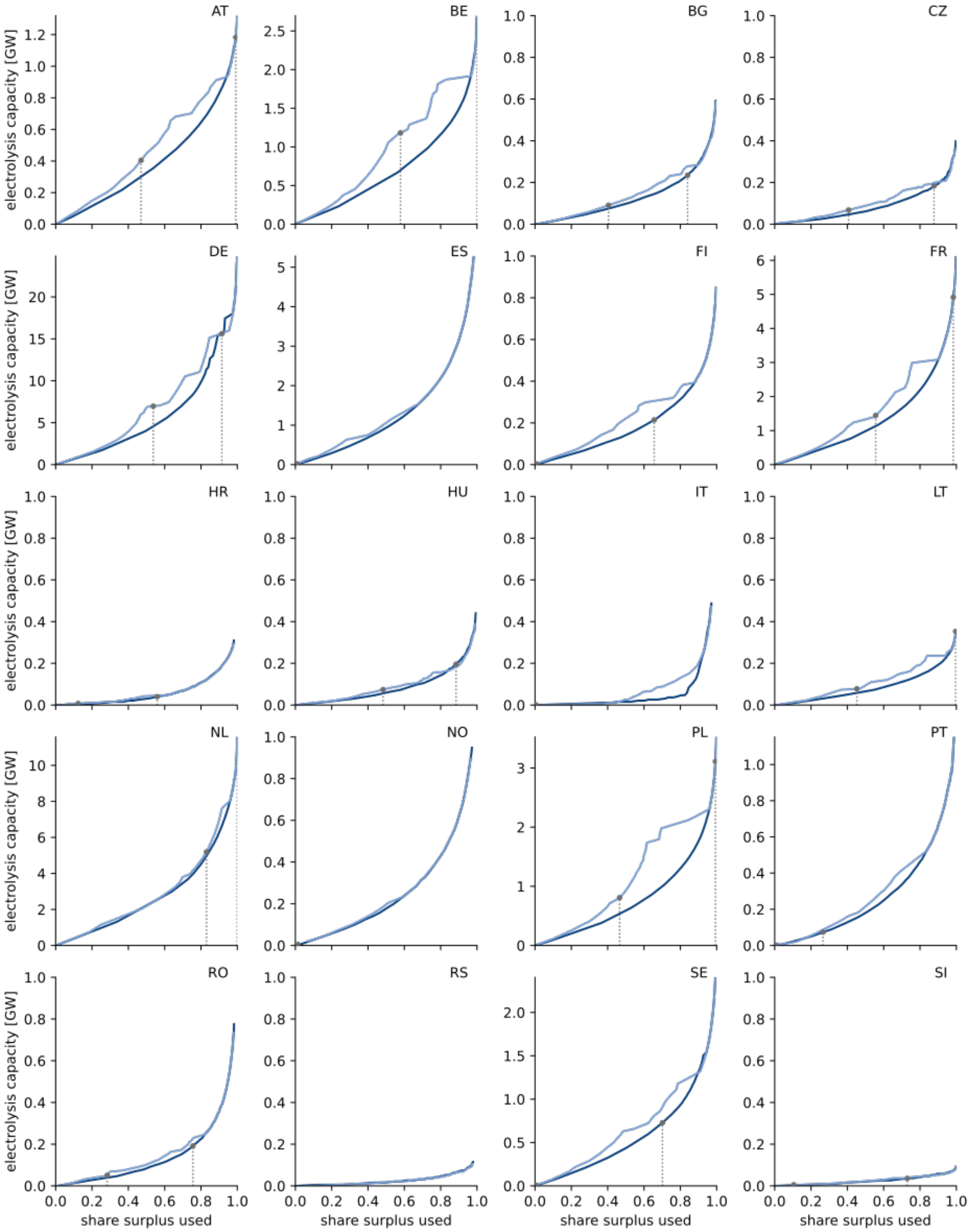

Figure S8. Country-level electrolysis capacity in the fuel-saving scenario (dark blue) and the fuel-replacing scenario (light blue) depending on the utilization of the available surplus electricity. The grey dotted line indicates the capacity and share of surplus electricity used when the levelized cost corresponds to the average levelized cost for fossil hydrogen production reported in 2023 (3.5 €/kg$_{H2}$) [1].

S4.3. Electrolysis capacity factor as a function of the utilization rate of surplus electricity

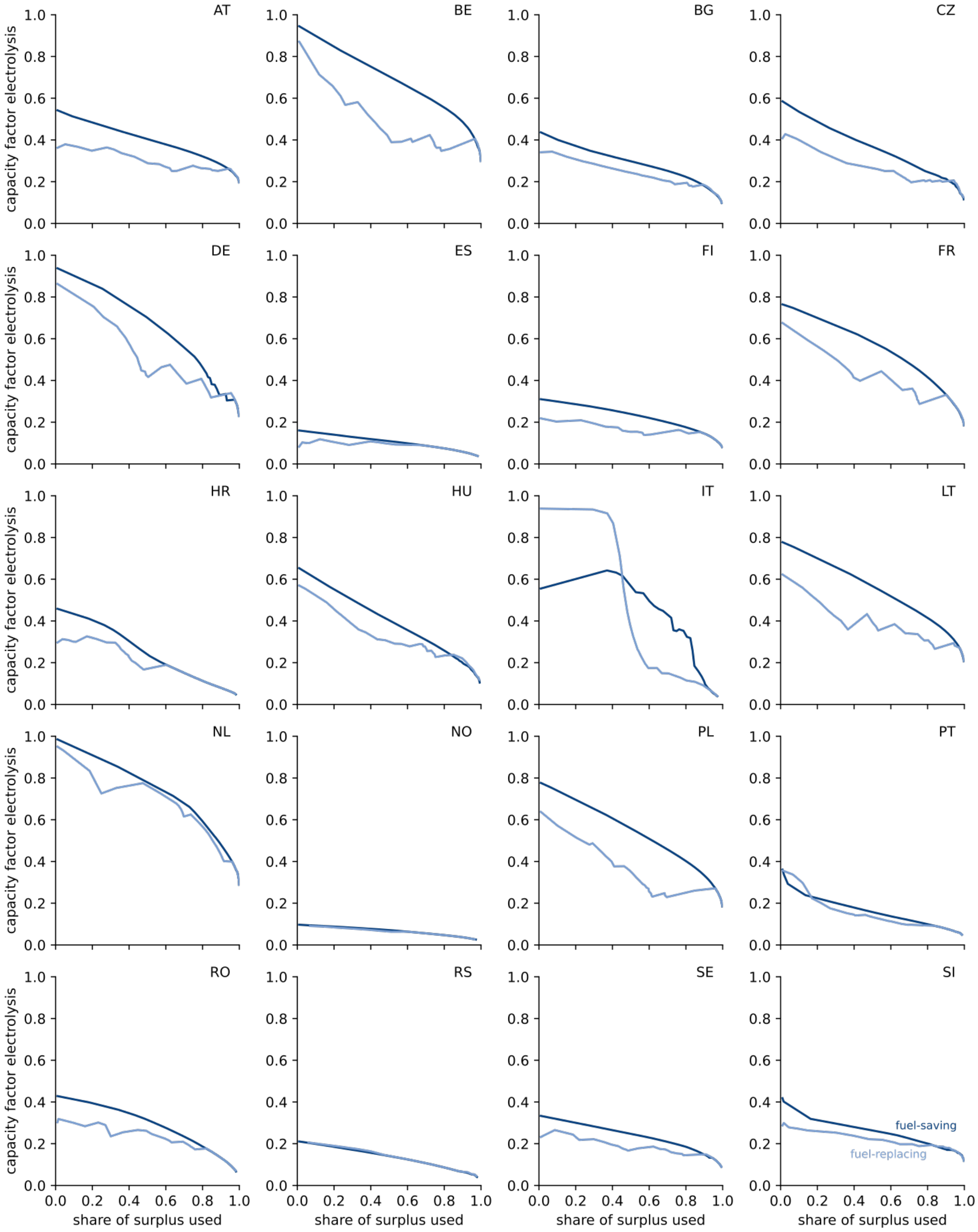

Figure S9. Country-level electrolysis capacity factor in the fuel-saving scenario (dark blue) and the fuel-replacing scenario (light blue) depending on the utilization of the available surplus electricity.

S4.4. Hydrogen storage capacity of the utilization rate of surplus electricity

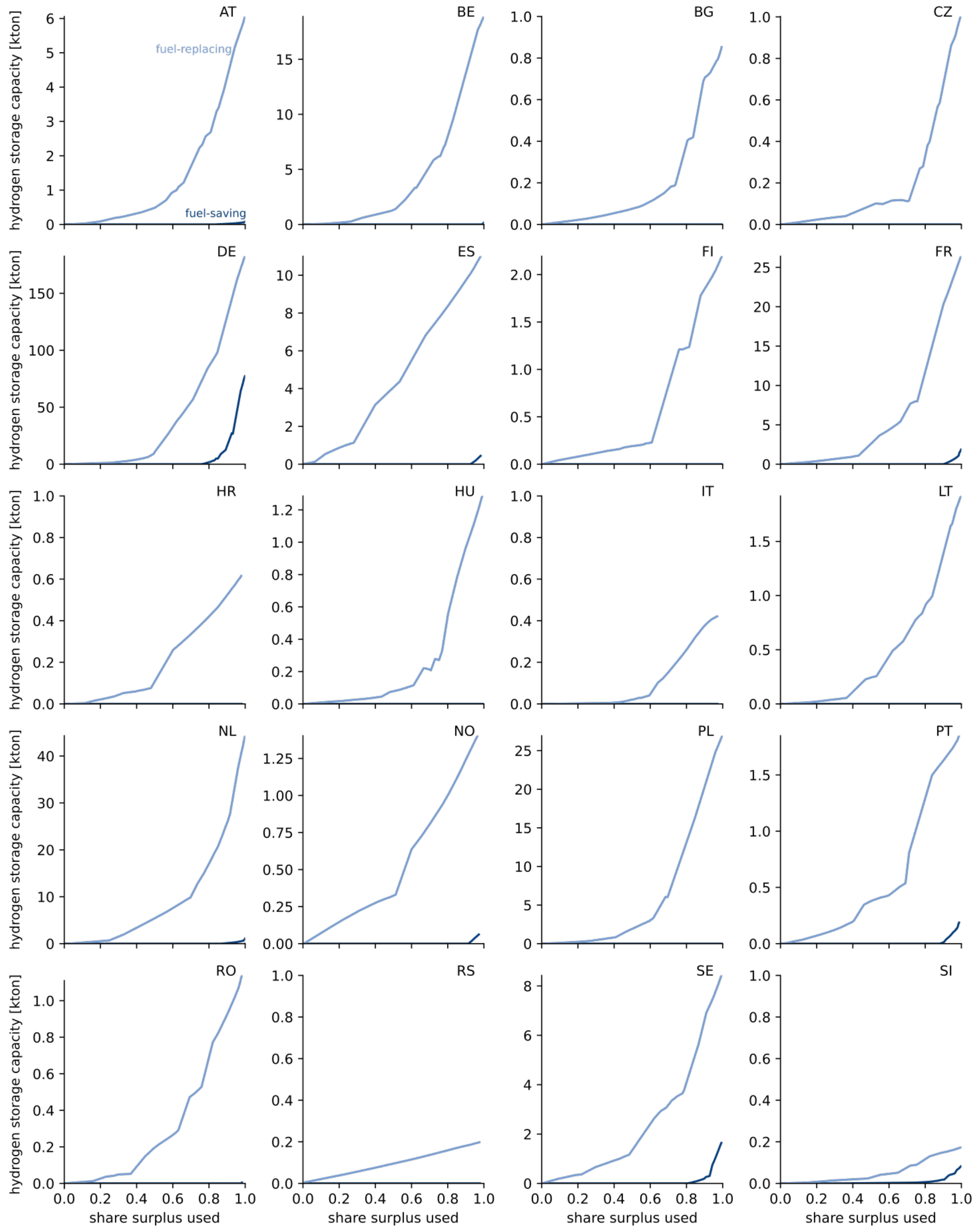

Figure S10. Country-level hydrogen storage capacity in the fuel-saving scenario (dark blue) and the fuel-replacing scenario (light blue) depending on the utilization of the available surplus electricity.

S4.5. Battery storage capacity of the utilization rate of surplus electricity

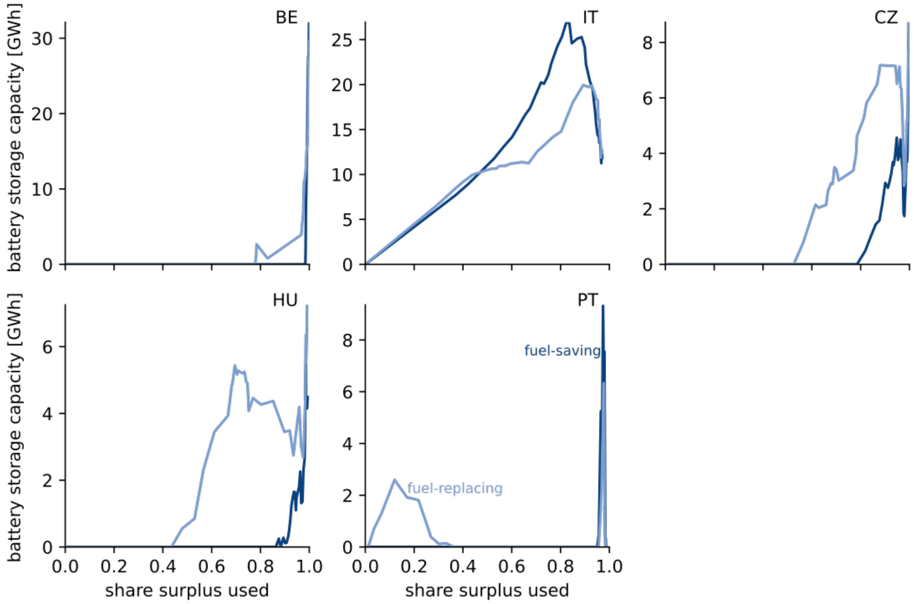

Figure S11. Country-level battery storage capacity in the fuel-saving scenario (dark blue) and the fuel-replacing scenario (light blue) depending on the utilization of the available surplus electricity. As increasing amounts of surplus renewable electricity are utilized to produce hydrogen, it may prove more cost-effective to reduce battery capacity and increase electrolyzer capacity instead of expanding battery storage capacity depending on the profile of the time series of the surplus electricity.

S4.6. Electrolysis capacity for countries with export potential for hydrogen from surplus electricity

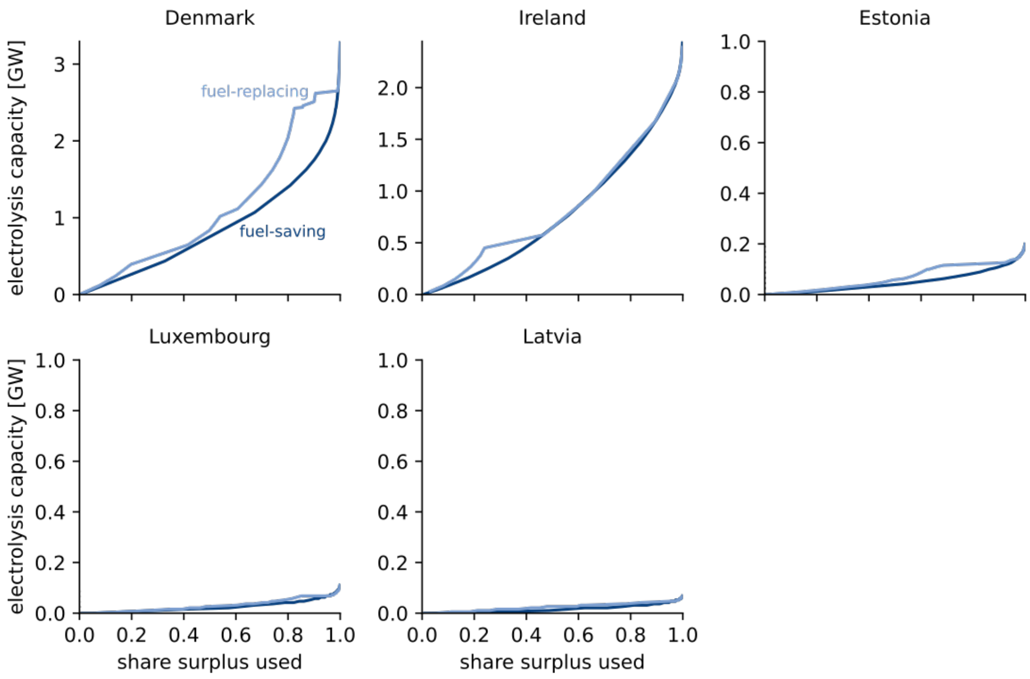

Figure S12. Electrolysis capacity for countries where hydrogen production from surplus electricity exceeds local demands, and which, thus, could export hydrogen to neighboring countries. Two supply scenarios are considered, fuel-saving scenario (dark blue, unconstrained supply) and fuel-replacing scenario (light blue, hourly constant supply).

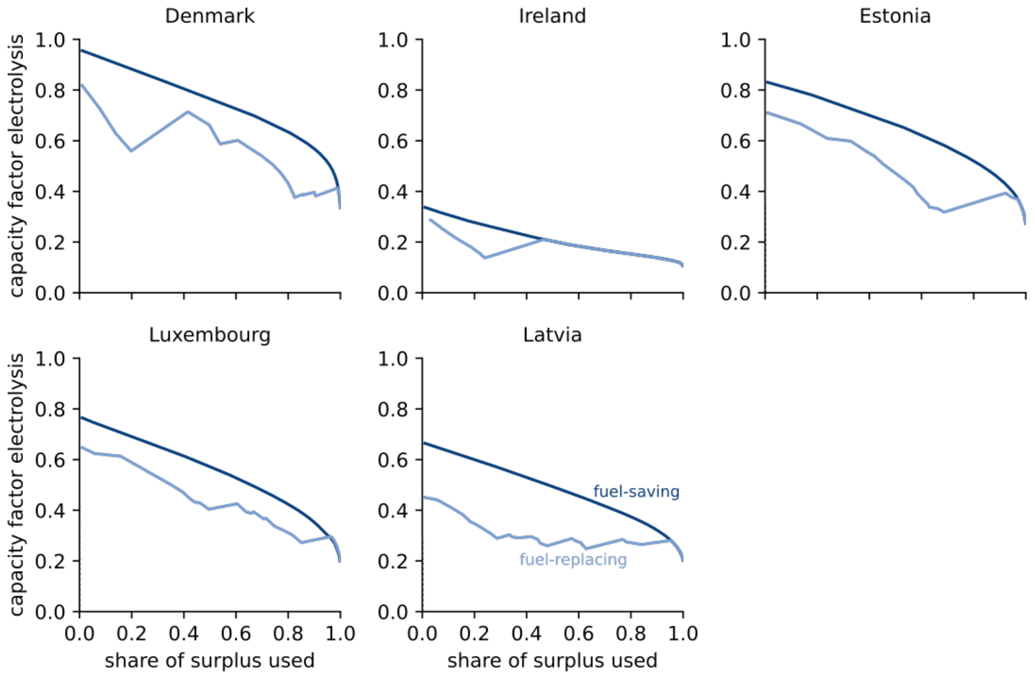

Figure S13. Electrolysis capacity factor for countries where green hydrogen production exceeds local demands, and which, thus, could export green hydrogen to neighboring countries. Two supply scenarios are considered, fuel-saving scenario (dark blue, unconstrained supply) and fuel-replacing scenario (light blue, hourly constant supply).

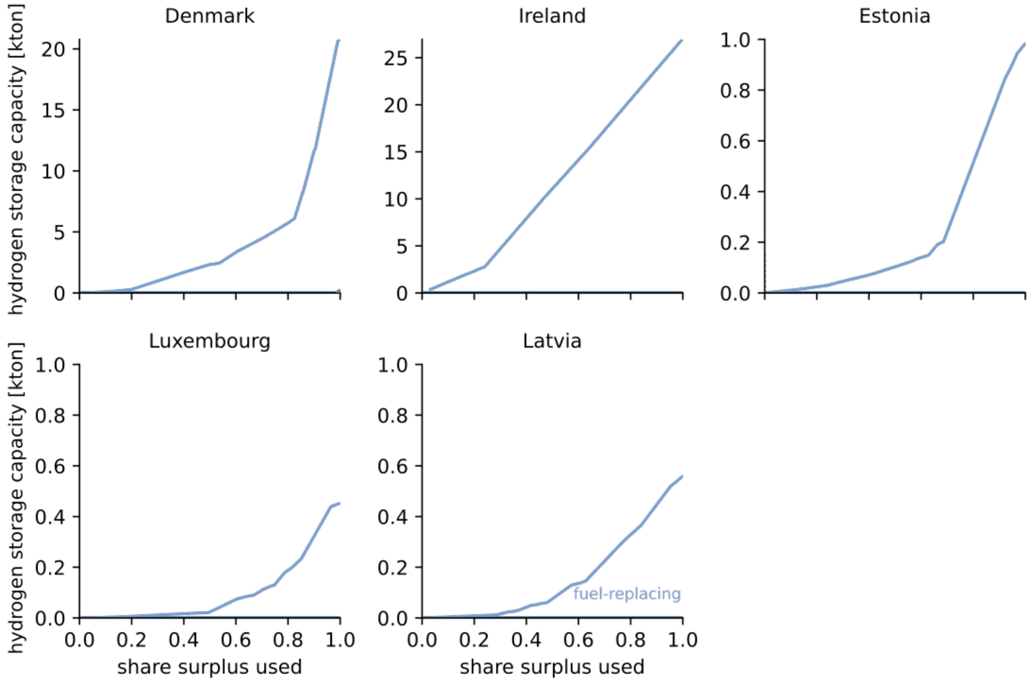

Figure S14. Hydrogen storage capacity for countries where green hydrogen production exceeds local demands, and which, thus, could export green hydrogen to neighboring countries. Two supply scenarios are considered, fuel-saving scenario (dark blue, unconstrained supply) and fuel-replacing scenario (light blue, hourly constant supply).

S4.7. Storage cost sensitivity

We investigate the sensitivity of our results in terms of the battery storage cost assumptions. Figure S15 shows the change in the LCOH if battery storage cost increases (blue) or decreases (red) by 25%, respectively. The LCOH in Italy (IT) is affected most by changes in the battery storage costs. Compared to other countries, the variability of the surplus electricity in Italy is high (Figure S3c), making the addition of battery storage capacity cost-effective to balance its intermittent availability. Conversely, a cost reduction enables the installation of slightly larger battery storage capacity whilst reducing the LCOH. However, for all other countries, the addition of battery storage capacity remains cost-ineffective, and the LCOH remains small in most cases (<5%).

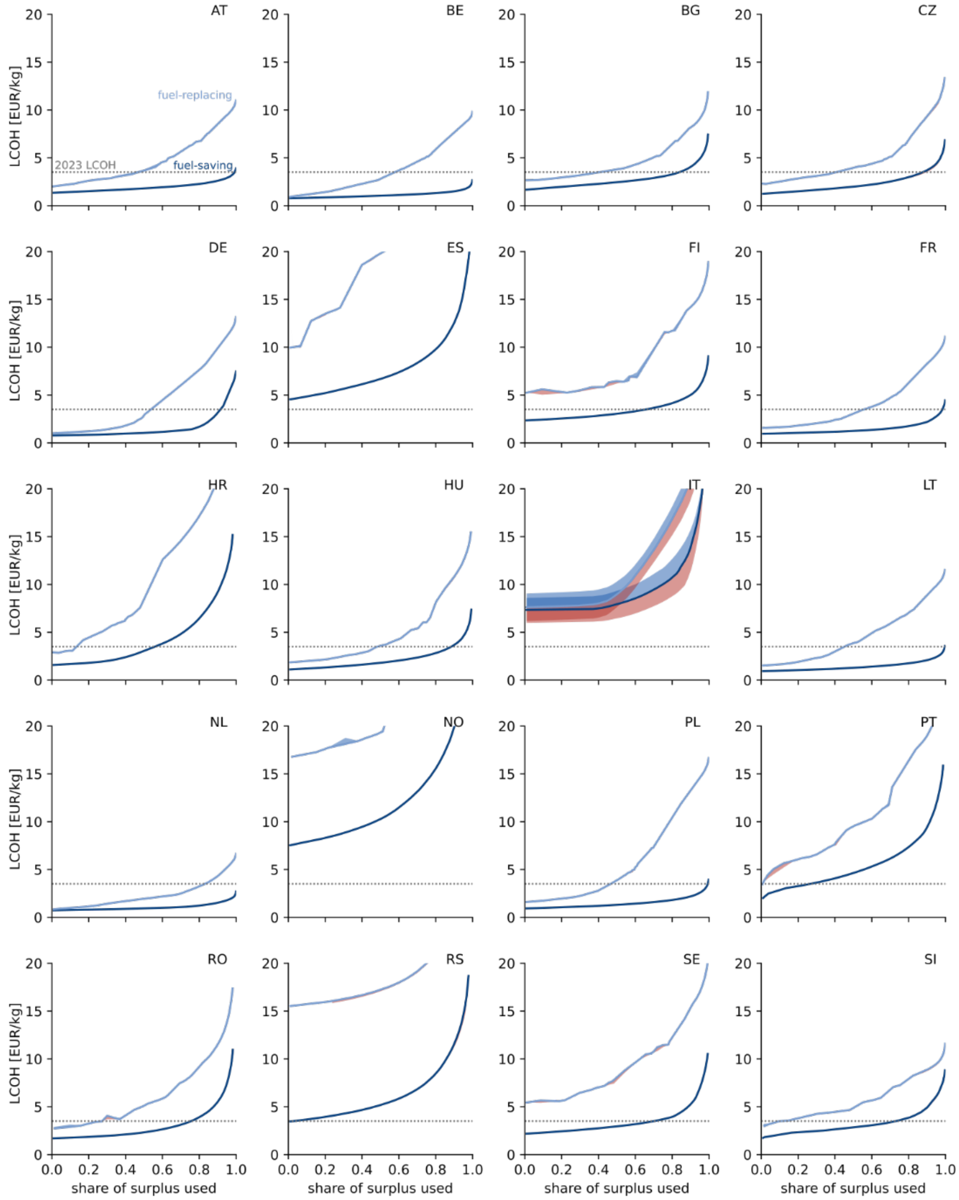

Figure S15. Country-level levelized cost of hydrogen (LCOH) in the fuel-saving scenario (dark blue) and the fuel-replacing scenario (light blue), depending on the utilization of the available surplus electricity. The grey dotted line indicates the country-level LCOH reported in 2022. The blue and red areas indicate the resulting change in the LCOH if storage costs increase or decrease by 25%, respectively.

References

1. Fonseca J, Muron M, Pawelec G, Yovchen IP, Kuhn M, Fraile D, et al. Clean Hydrogen Monitor 2023. Hydrogen Europe; 2023. Available from: https://hydrogeneurope.eu/wp-content/uploads/2023/10/Clean_Hydrogen_Monitor_11-2023_DIGITAL.pdf (accessed 2023-11-22)

2. Georg Bilcic, Samuel Scroggins. Lazard's Levelized Cost of Energy +. Lazard; 2023. Available from: https://www.lazard.com/media/2ozoovyg/lazards-lcoeplus-april-2023.pdf (accessed 2024-04-24)

3. Papadias DD, Ahluwalia RK. Bulk storage of hydrogen. Int J Hydrog Energy. 2021-10-11;46(70):34527–41.

4. European Hydrogen Observatory. Hydrogen Demand. 2022. Available from: https://observatory.clean-hydrogen.europa.eu/hydrogen-landscape/end-use/hydrogen-demand (accessed 2024-06-18)

5. eurostat. NUTS - Nomenclature of territorial units for statistics. 2021. Available from: https://ec.europa.eu/eurostat/web/nuts/overview (accessed 2024-04-07)

6. The ENTSO-E Transparency Platform – A review of Europe's most ambitious electricity data platform - ScienceDirect. Available from: https://www.sciencedirect.com/science/article/pii/S0306261918306068 (accessed 2024-06-03)

7. ENTSO-E. ENTSO-E Transparency. 2024. ENTSO-E Transparency Platform. Available from: https://transparency.entsoe.eu/ (accessed 2024-02-29)
